# ^177^Lu-labeled DOTA-EB-RGD as a candidate for thyroid cancer therapy

**DOI:** 10.1172/jci.insight.200594

**Published:** 2026-10-08

**Authors:** Sonam Kumari, Rhitajit Sarkar, Zhantong Wang, Shilpa Thakur, Laura Abaandou, Oksana Gavrilova, Huiyan Lu, Noha Behairy, Lixin Lang, Dale Kiesewetter, Vasyl Vasko, Joanna Klubo-Gwiezdzinska

**Affiliations:** 1 Metabolic Diseases Branch, National Institute of Diabetes and Digestive and Kidney Diseases,; 2 Mouse Metabolism Core, National Institute of Diabetes and Digestive and Kidney Diseases, and; 3 Molecular Tracer and Imaging Core Facility, National Institute of Biomedical Imaging and Bioengineering, NIH, Bethesda, Maryland, USA.; 4 Department of Pediatric Endocrinology, Uniformed Services University of the Health Sciences, Bethesda, Maryland, USA.

**Keywords:** Endocrinology, Oncology, Thyroid disease

## Abstract

Integrin α_V_β_3_, a transmembrane receptor involved in tumor growth and metastasis, specifically recognizes and binds proteins with the Arg-Gly-Asp (RGD) peptide sequence. Using mRNA and/or protein expression data from 496 thyroid cancer (TC) samples in The Cancer Genome Atlas, 14 TC cell lines, and 70 TC and 10 normal thyroid tissues, we found that papillary TC exhibits the highest α_V_β_3_ integrin expression. We then evaluated the therapeutic efficacy of a radiolabeled RGD analog, ^177^Lu-DOTA-EB-cRGDfK, in TC. Genetic knockout and overexpression of α_V_β_3_ in TC cell lines confirmed the target specificity of ^177^Lu-DOTA-EB-cRGDfK. In mouse xenograft models established from human TC cell lines with high α_V_β_3_ expression, ^177^Lu-DOTA-EB-cRGDfK demonstrated superior antitumor efficacy compared with standard-of-care lenvatinib and placebo. However, no synergistic benefit was observed with combination therapy. Biodistribution studies identified the kidneys as the dose-limiting organ. RNA-seq analysis of resected tumors demonstrated that ^177^Lu-DOTA-EB-cRGDfK induced a type I IFN response, characterized by upregulation of *IFI6*, *IFIT1-2*, and *MX1*, compared with both lenvatinib-treated tumors and placebo. These findings identify α_V_β_3_ integrin as a promising therapeutic target in a subset of TCs and demonstrate that ^177^Lu-DOTA-EB-cRGDfK exhibits superior efficacy to lenvatinib, supporting its potential clinical translation for progressive TC refractory to standard therapies.

## Graphical abstract

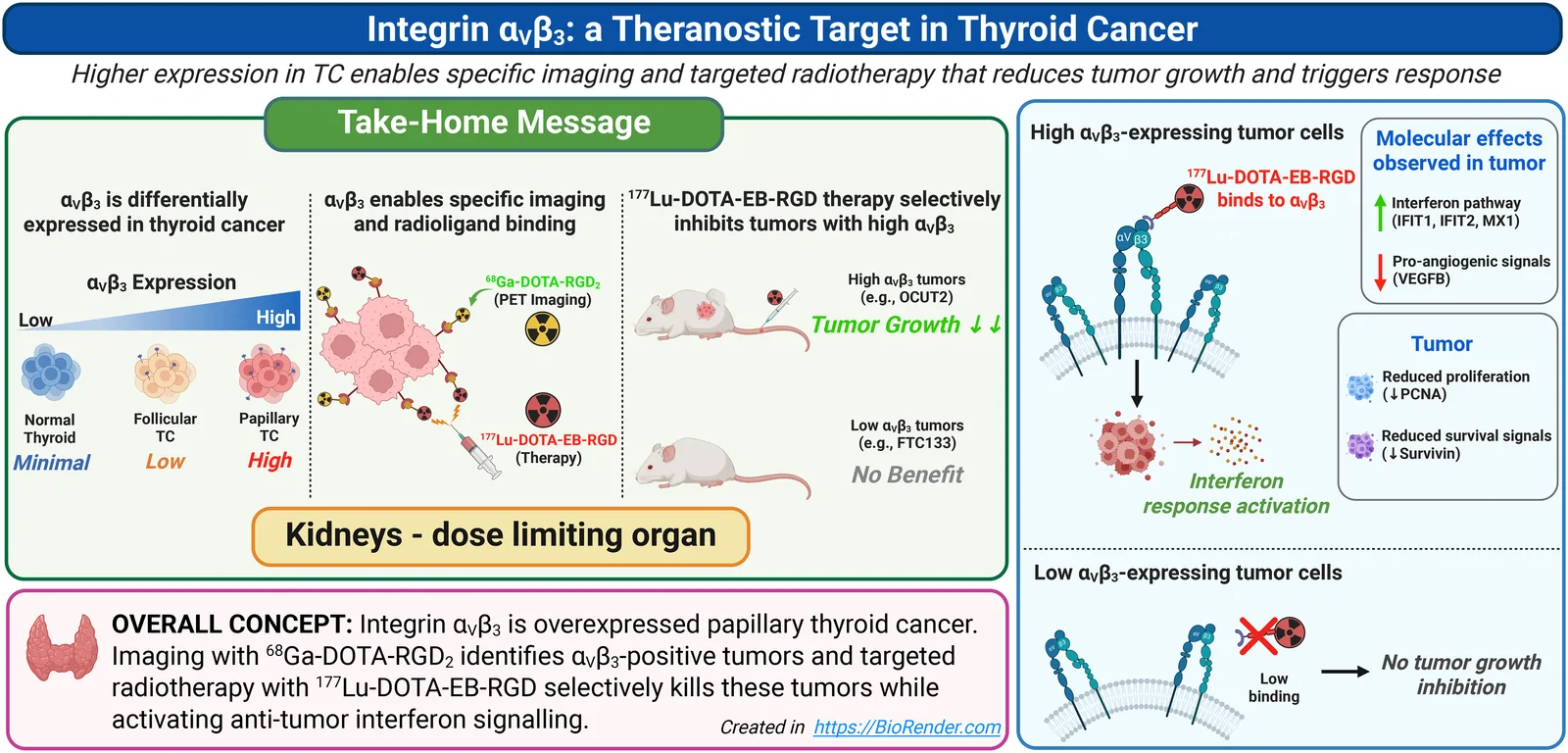

## Introduction

Thyroid carcinoma (TC) is categorized by the World Health Organization into *BRAF*-like, *RAS*-like, and non-*BRAF/RAS*-like tumors based on its histology, differentiation, and molecular signature (1). The standard treatment for aggressive TC consists of the complete removal of thyroid gland and radioactive iodine (RAI) treatment (2). However, tumors become refractory to standard therapy in approximately 22% of patients (3, 4). Several studies have suggested that the survival rate of non–RAI-responsive TC patients is less than 3.5 years (5, 6). The use of tyrosine kinase inhibitors (TKIs), such as lenvatinib for treatment of advanced RAI refractory TC, is driven by its growth inhibitory effects elicited via multiple targets such as fibroblast growth factor receptors 1–4 (FGFR-1–4), vascular endothelial growth factor receptors 1–3 (VEGFR1–3), c-kit, RET, and platelet-derived growth factor receptor a (PDGFRa). However, therapy with TKIs leads only to transient responses (7, 8). Therefore, it is imperative to devise new therapeutic strategies to overcome these challenges.

Integrins are transmembrane receptors that can identify and bind to extracellular matrix proteins. The binding of integrins to their respective ligands leads to several structural, as well as signaling, changes in cells, including alterations in gene expression. Moreover, different levels of crosstalk exist between integrin-mediated signaling and signaling downstream of growth factors (9). A subtype of integrins (α_V_β_3_) recognizes Arg-Gly-Asp (RGD) peptide motifs, which have a role in neovascularization and development of different cancers (10).

Radiolabeled RGD peptides are utilized as radiotracers for targeted tumor imaging and therapy. In fact, there are preliminary data revealing that the radiolabeled α_V_β_3_ agonist ^68^Ga-DOTA-RGD_2_ could be successfully used for positron emission tomography/computed tomography (PET/CT) imaging in patients with radioiodine-refractory TC. These findings suggest that targeting α_V_β_3_ with RGD-based ligands radiolabeled with β emitters such as lutetium-177 (^177^Lu) may have therapeutic potential in TC. However, given the relatively short half-life of DOTA-RGD (11, 12), we believe that the therapeutic efficacy of ^177^Lu-labeled RGD peptides can be improved by conjugating the Evans Blue (EB) moiety to the cyclic form of RGD peptide that extends its half-life and therapeutic efficacy (13). Therefore, the goal of this study was to assess the expression of α_V_β_3_ in different histological and molecular subtypes of TC and to test the therapeutic efficacy of the radiolabeled integrin α_V_β_3_ targeting agent, ^177^Lu-DOTA-EB-cRGDfK, in the therapy of TC in mouse xenografts.

## Results

### Expression of α_V_β_3_ integrins in BRAF-like and RAS-like tumors.

We utilized the publicly available database, The Cancer Genome Atlas (TCGA), consisting of mRNA expression profiles of 496 TC tissues to evaluate the association between *BRAF-RAS* scoring (BRS) and the expression of integrins α_V_ and β_3_. We observed that there was a negative correlation between mRNA expression of α_V_ and BRS (*r* = –0.5, *P* < 0.001) (Figure 1A). In addition, we found a low negative correlation between mRNA expression of β_3_ and BRS (*r* = –0.27, *P* < 0.001) (Figure 1B), suggesting that expression of α_V_ and β_3_ integrins might be higher in *BRAF*-like tumors. Consistently with this result, RNA-seq data in selected samples harboring the *BRAFV600E* mutation (*n* = 143) and *HRAS/NRAS/KRAS* pathogenic variants (*n* = 49) showed that the expression of α_V_ was significantly higher (*P* = 0.001) in *BRAFV600E*-driven TC as compared with *RAS*-driven tumors (Figure 1C), but there was no statistically significant difference in β3 expression between *BRAF*-like and *RAS*-like tumors (*P* = 0.07) (Figure 1D). To summarize, mRNA expression of α_V_ is higher in *BRAF*-like than *RAS*-driven tumors, but its dimer’s partner β_3_ is not significantly associated with *BRAF/RAS* transcriptome profile. Therefore, we next focused on the analysis of α_V_β_3_ protein dimers in TC.

### Papillary TC has higher α_V_β_3_ expression compared with follicular TC and normal thyroid.

Human tissue microarrays including 70 TC (papillary thyroid carcinoma [PTC] = 44, follicular thyroid carcinoma [FTC] = 20, poorly differentiated thyroid carcinoma [PDTC] = 6) and 10 normal thyroid samples were employed for immunohistochemistry. Higher α_V_β_3_ expression was found in PTC compared with FTC (*P* = 0.03), and between PTC and normal thyroid (*P* < 0.001) (Figure 1, E and F). We next modeled the observed variable expression of α_V_β_3_ in vitro in TC cell lines.

### Differential expression of α_V_β_3_ in TC cell lines.

We analyzed the mRNA expression of 24 genes, including integrins α_V_β_3_ and thyroid-specific genes (thyrotropin receptor [*TSHR*], thyroglobulin [*TG*], thyroid peroxidase [*TPO*], thyroid hormone receptor α [*THRA*], and *THRB*) in 13 TC cell lines and the normal thyroid cell line (HTori-3) (Figure 2A). As expected (14), TC cell lines were characterized by a significantly reduced expression of thyroid-specific genes compared with normal thyrocytes. We found a differential mRNA expression of α_V_β_3_ integrin in TC cell lines (Figure 2A). While the mRNA level of α_V_ (*ITGAV*) expression was relatively comparable between the cell lines and normal thyroid, the highest β_3_ (*ITGB3*) integrin expression was observed in TC cell lines driven by the *BRAFV600E* oncogenic mutation (K1, OCUT2, SW1736), *MKRN1-BRAF* fusion (THJ16T), or *CCDC6-RET* fusion (TPC1).

Relative mRNA expression patterns were confirmed at the protein level by immunoblotting and cell surface immunofluorescent staining, and 6 TC cell lines with variable α_V_β_3_ expression were selected for further analyses. There was no statistically significant correlation between α_V_β_3_ dimers and *BRAF/RAS* status of TC cell lines (*r* = –0.34, *P* = 0.51, Figure 2, B–D), despite a moderate-to-high correlation of individual monomers α_V_ (*r* = –0.7, *P* = 0.12, Supplemental Figure 1, A–C; supplemental material available online with this article; https://doi.org/10.1172/jci.insight.200594DS1), and β_3_ (*r* = –0.61, *P* = 0.19, Supplemental Figure 1, D–F).

Taken together, these data suggest no association between α_V_β_3_ dimer expression and *BRAF*-like or *RAS*-like transcriptome profile. However, we have established a preclinical model of differential α_V_β_3_ expression in human TC.

### Cell surface expression of α_V_β_3_ integrin receptors.

To analyze cell surface expression of α_V_β_3_, we performed flow cytometry on cell lines characterized by high (OCUT2) and low (FTC133) overall α_V_β_3_ expression. The dot plots (Figure 3A) show a higher percentage of α_V_β_3_-positive cells in OCUT2 (99.7%) than in FTC133 (63.2%), as is corroborated by the staggered overlay of the univariate histogram (Figure 3B) for the detector channel. Moreover, comparison of the mean fluorescence intensity (MFI) showed that OCUT2 is characterized by a 2.6-fold higher (*P* < 0.001) expression of α_V_β_3_ on the cell surface as compared with FTC133 (Figure 3C). When *ITGB3* was knocked out in OCUT2 (OCUT-β3KO), the cell surface expression of α_V_β_3_ dimer was not detected (Figure 3D). The corresponding scatter dot plot for the MFI supported the observation, showing a 10-fold decrease to a near-negligible level of α_V_β_3_ in OCUT-β3KO cells (*P* < 0.0001). On the other hand, with adenoviral overexpression of *ITGAV* and *ITGB3* in the FTC133 cell line (FTC133-α_V_β_3_^+^, Supplemental Figure 2), the α_V_β_3_ cell surface expression increased, as shown in the staggered histogram (Figure 3E) and in the adjoining dot plot, with a 4-fold increase compared with parental FTC133 (*P* < 0.0001).

### Saturation radioligand binding to α_V_β_3_ integrin receptors.

Consistently, radioligand binding assays to the α_V_β_3_ receptors, showed a significantly higher maximum binding sites (B_max_) in OCUT2 cells as compared with FTC133 cells (mean ± SEM of 422.5 ± 49.4 and 88.1 ±13.9 fmol, respectively, *P* = 0.016; Figure 4, A and B), with the former having a significantly higher binding affinity (K_d_) value (OCUT2 344.1 ± 57.6 nM vs. FTC133 97.4 ± 10.0 nM, *P* = 0.047). Accordingly, we have identified TC cell line models with high and low α_V_β_3_ cell surface expression for testing in vivo. In order to identify the specificity of the RGD peptide to the cell surface dimer α_V_β_3_ receptor, saturation binding of ^68^Ga-DOTA-RGD_2_ in OCUT-β3KO cells shows (Figure 4A) near-negligible affinity for the ligand (B_max_ 11.3 ± 1.7 fmol) compared with the parental OCUT2 (extra-sum-of-squares *F* test, *F*(2,176) = 907.8, *P* < 0.0001). Saturation binding curves differed significantly between FTC133 and FTC133-α_V_β_3_^+^ cells (extra-sum-of-squares *F* test, *F*(2,116)=206.6, *P* < 0.0001). Nonlinear regression revealed a marked increase in B_max_ in FTC133-α_V_β_3_^+^ cells (206.6 ± 24.5 vs. 88.1 ±13.9 fmol/mg, *P* < 0.0001), whereas K_d_ values were similar (107.1 ± 11.8 vs. 97.4 ± 10 nM, *P* = 0.58), as shown in Figure 4B.

Taken together, these data confirm the specificity of ^68^Ga-DOTA-RGD_2_ binding to α_V_β_3_ integrin.

### ^177^Lu-DOTA-EB-RGD reduces tumor growth in TC tumor models with high α_V_β_3_ integrin expression.

We compared the therapeutic potential of radiolabeled RGD peptides with lenvatinib, the existing standard of care, in vivo using our established mouse models (15, 16). We selected subcutaneous and/or metastatic models with variable α_V_β_3_ expression and binding capacity; OCUT2 served as a model with high expression and high binding capacity and FTC133 as a model with low α_V_β_3_ expression and low B_max_. Mixed-effects model-based analysis of tumor growth velocity revealed that treatment with ^177^Lu-DOTA-EB-RGD (^177^Lu) led to a significant reduction in the tumor growth over time as compared with placebo (*P* < 0.001) and lenvatinib alone (*P* < 0.001, Figure 5A). The combination therapy (^177^Lu+L) resulted in decreased tumor growth compared with monotherapy with lenvatinib (*P* < 0.001) but had a similar effect to ^177^Lu monotherapy (*P* = 0.79) (Figure 5, A and C). In addition, PET scanning revealed that the uptake of ^68^Ga-DOTA-RGD_2_ was significantly reduced due to a smaller tumor volume in mice exposed to combination therapy (^177^Lu+L, maximum standard uptake value [SUV_max_] 0.75 ± 0.11) as compared with treatment with placebo (SUV_max_ 1.12 ± 0.11; *P* = 0.001) as well as lenvatinib (0.98 ± 0.21; *P* = 0.045) (Figure 5B).

There was no treatment-associated difference in ^68^Ga-DOTA-RGD_2_ distribution in normal organs apart from higher variability in heart exposure in ^177^Lu monotherapy as compared with placebo (Figure 5D).

Ex vivo biodistribution studies in ^177^Lu monotherapy and ^177^Lu+L revealed that the kidneys had a similar radiation exposure to the tumors, while exposure to the heart (*P* < 0.0001) and liver (*P* < 0.001) was significantly lower (Figure 5E). These data suggest that kidneys might be the dose-limiting organ for bench-to-bedside translation.

RNA-seq analysis of resected tumors demonstrated that ^177^Lu either in monotherapy or combination therapy induced a type I IFN response, characterized by upregulation of IFN-α–inducible protein 6 (*IFI6*) that was the only transcript passing the false discovery rate (FDR) cutoff of *q* less than 0.05 (log_2_FC 3.5, *P* < 0.001, *q* = 0.01). Therefore, given the exploratory nature of the analysis and the limited sample size, genes with unadjusted *P* of less than 0.05 and |log_2_FC| of 1 or greater were considered candidates for further validation. Consistently, other IFN response element genes were also significantly upregulated IFN-induced protein with tetratricopeptide repeats 1 (*IFIT1*), *IFIT2*, and MX dynamin-like GTPase 1 (*MX1*), compared with both placebo- and lenvatinib-treated tumors (Supplemental Figure 3, A, C, and D, Supplemental Figure 4, and Supporting Data Values; unadjusted *P* < 0.05, and |log_2_FC| ≥ 1). Treatment with ^177^Lu was also associated with reduced expression of the proangiogenic factor vascular endothelial growth factor β (VEGFB) (Supplemental Figure 3A and Supporting Data Values), whereas lenvatinib predominantly suppressed epithelial-mesenchymal transition–associated (EMT-associated) and invasion-associated genes, including fibronectin 1 (*FN1*), cluster of differentiation 44 (*CD44*), *MET* protooncogene, catenin β1 (*CTNNB1*), and matrix metallopeptidase 2 (*MMP2*) (Supplemental Figure 3B and Supporting Data Values). No significant changes in α_V_ and β_3_ integrin mRNA expression were observed in mice treated with ^177^Lu monotherapy, whereas lenvatinib monotherapy was associated with reduced expression of integrin-associated adhesion and invasion genes, including *ITGAV*, *ITGB5*, *FN1*, *CD44*, and *MMP2* (Supplemental Figure 3B and Supporting Data Values). However, downstream focal adhesion signaling components, including focal adhesion kinase (FAK, encoded by *PTK2*), *SRC* protooncogene, paxillin (*PXN*), and vinculin (*VCL*), did not exhibit a significant differential expression across treatment groups. Consistently, the protein expression of α_V_β_3_ dimers was significantly reduced in lenvatinib (3.0 ± 0.82 vs. 8.86 ± 2.27, *P* = 0.001) and L+^177^Lu combination therapy (3.5 ± 01.52 vs. 8.86 ± 2.27, *P* = 0.01), as compared with placebo (Figure 6). However, its decreased expression was also observed in ^177^Lu monotherapy (3.33 ± 0.52 vs. 8.86 ± 2.27, *P* = 0.01), despite no detectable changes in integrin transcript levels. This finding suggests that ^177^Lu treatment may have preferentially eliminated clones with high integrin expression. Furthermore, immunostaining revealed uniform FAK expression across treatment groups (Supplemental Figure 5, A and B), indicating that cell-matrix anchorage signaling was not differentially affected. Additional downstream targets of integrin signaling, pan-AKT, p-ERK1/2, and p-MEK, were also not significantly affected in any of the treatment groups. Similarly, no differential expression was found for tumor-suppressing PTEN in any of the studied groups (Supplemental Figure 5, A and B). Collectively, these findings suggest that the therapeutic activity of ^177^Lu-DOTA-EB-RGD is not mediated by major transcriptional downregulation of α_V_β_3_ integrin.

Immunostaining for proliferation and anti-apoptotic markers (Figure 6A) revealed that compared with placebo, proliferation marker PCNA was significantly reduced in the tumors subjected to combination therapy with ^177^Lu+L (median score 2.5 [IQR 2–3.75] vs. 9 [IQR 6–12], *P* = 0.001) and monotherapy with lenvatinib (median score 2 [IQR 2–4] vs. 9 [IQR 6–12], *P* < 0.001), while there was a slight, statistically nonsignificant decrease in the proliferation marker with ^177^Lu alone as compared with placebo (median score 6 [IQR 5.25–6] vs. 9 [IQR 6–12], *P* = 0.47). Compared with tumors exposed to placebo, the cancer cells’ survival marker survivin was significantly reduced in ^177^Lu combination therapy (score 3.83 ± 2.4 vs. 7.71 ± 3.14, *P* = 0.03) and ^177^Lu monotherapy (score 2.83 ± 1.94 vs. 7.71 ± 3.14, *P* = 0.01) (Figure 6B). However, expression of key apoptotic regulators BAX and BCL2 (Supplemental Figure 6, A and B) maintained steady, localized cytoplasmic positivity across all cohorts, indicating that neither mono nor combination treatments significantly altered baseline proapoptotic signals compared with placebo control.

In contrast, the luciferase-tagged FTC133 induced lung metastatic mouse model, characterized by a low α_v_β_3_ expression, did not identify metastatic lesions with ^68^Ga-DOTA-RGD_2_ PET imaging despite their presence being documented by IVIS imaging (Supplemental Figure 7A), consistent with low to no α_V_β_3_ expression in metastatic lesions. This was associated with no reduction in tumor growth in response to ^177^Lu, since no significant difference in tumor burden via bioluminescent counts from the IVIS imaging studies was observed across all treatment groups compared to placebo (Supplemental Figure 7, A and B).

Taken together, these in vivo data indicate that treatment with ^177^Lu, either as monotherapy or in combination with lenvatinib, is effective in reducing the growth of TC characterized by high α_v_β_3_ expression, and its effects are associated with eliciting a type I IFN signaling, increased prosurvival signaling, and reduced proliferation in vivo.

## Discussion

In this study, we demonstrated that integrin α_V_β_3_ is upregulated in a subset of TC, particularly in the most common histological type, PTC, and that ^68^Ga-labeled RGD can be used for PET imaging of tumors characterized by a high α_V_β_3_ expression. While PTC is characterized by the highest α_V_β_3_ expression, there is no association between its expression and *BRAF*-like or *RAS*-like status of TC. We next demonstrated that the radiolabeled α_V_β_3_ analog ^177^Lu-DOTA-EB-RGD targets α_V_β_3_ in TC, leading to tumor growth inhibition in models characterized by high α_V_β_3_ expression, but not in models characterized by low α_V_β_3_ expression. The therapeutic efficacy was associated with eliciting a type I IFN signaling, inhibition of proliferation and decreased TC cell survival, as well as with decreased α_V_β_3_ expression in treated tumors. Decreased proliferation and diminished prosurvival markers are consistent with the growth inhibitory potency of therapy with ^177^Lu-DOTA-EB-RGD. Because no changes in α_V_β_3_ were observed at the transcriptional level, and no changes in activation of its downstream targets have been observed on a protein level, the lower α_V_β_3_ protein expression in treated tumors may reflect selective elimination of α_V_β_3_-high tumor cells, resulting in enrichment of residual cells with lower receptor expression. In addition, reduced tumor cellularity, stromal remodeling, may also contribute to the observed decrease in protein expression.

Our preliminary transcriptomic data showing induction of IFN-stimulated genes after ^177^Lu-DOTA-EB-RGD are consistent with emerging evidence that ^177^Lu-based radiopharmaceutical therapies can activate a STING-dependent type I IFN program in tumor cells associated with an increased intratumoral CD8^+^/Treg ratio (17). Furthermore, ^177^Lu-labeled ligands targeting different protein receptors, including ^177^Lu-iPD-L1, have been shown to remodel the tumor immune microenvironment, enhance immune effector functions, and augment antitumor immunity (18). However, activation of IFN-stimulated genes in our model as potential mechanism of therapeutic efficacy contributing to treatment-associated immune modulation needs to be tested in an immunocompetent model.

Integrins have been targeted with radiolabeled ligands in various metastatic cancers such as gliomas and non–small cell lung cancer (NSCLC) (19–23). There are several clinical trials successfully utilizing PET/CT imaging for evaluation of α_V_β_3_-positive cells in lymphomas, prostate, lung, breast, or head and neck cancers, as well as clinical trials focused on the selection of patients that may benefit from integrin targeting peptide receptor radionuclide therapy (PRRT) based on PET/CT imaging (e.g., ClinicalTrials.gov NCT05549024, NCT06638034, and NCT05013086) (24, 25). There are also preclinical data in breast, prostate, and pancreatic cancers utilizing α_V_β_3_ ligands radiolabeled with α emitters (^225^Ac) showing a significant reduction in tumor burden (26–28). However, the application of this management strategy has not yet reached the clinical trial stage. There are also efforts to leverage integrins’ role in tumor neovascularization and combine RGD-analog-based therapy with antiangiogenic agents (24).

Interestingly, patients with metastatic TC have also been enrolled in small pilot studies focused on screening for α_V_β_3_ integrins using PET/CT (e.g., ClinicalTrials.gov NCT05549024 and NCT06638034). Parihar et al. evaluated the diagnostic accuracy of ^68^Ga-DOTA-RGD_2_ PET/CT in patients with radioiodine-refractory TC (RAIR-TC), validating the utilization of this radiotracer in the human population (12). The authors observed a high specific uptake of radiolabeled RGDs in metastatic lesions, inferring an optimal target for ^177^Lu-labeled RGDs for therapy. In a follow-up study, the same group presented a case report of a safe use of ^177^Lu-DOTA-RGD_2_ in a patient with metastatic RAIR-TC that resulted in improved pain control due to a reduction in size of one of the bone lesions located in the sternum at 4 months after therapy (29). However, the duration of such a response is unknown, as there are no follow-up data available. To optimize this therapeutic approach, patient selection may need to be guided by PET/CT imaging using ^68^Ga- or ^64^Cu-labeled α_V_β_3_ ligands to identify candidates for PRRT based on high tumor uptake. Given that our preclinical biodistribution studies identified the kidneys as the dose-limiting organ, PRRT may need to be accompanied by renoprotective amino acid infusions, similar to the standard practice used in PRRT targeting somatostatin receptors (30).

One of the limitations of RGD-based treatment is its limited stability, short half-life (24), and rapid renal clearance. Thus, it is crucial to improve its pharmacokinetics and pharmacodynamics. One strategy to overcome the drawback of low solution stability of RGD peptides is to cyclize the linear form of the RGD peptide (31, 32). Stability is enhanced by reducing conformational flexibility, leading to a lower susceptibility to proteolytic damage. Therefore, the cyclic form of RGD was used for therapy purposes, and RGD dimers for PET imaging. However, even the cyclic dimeric form has been characterized by a high K_d_ in our model. Therefore, in addition, in our study, cyclic RGD was chemically conjugated with an albumin-binding moiety-truncated Evans Blue (denoted as EB-RGD), leading to an increased half-life and elevated tumor uptake and tumor residence time (13, 33, 34). Zhao et al. utilized ^177^Lu-conjugated DOTA-EB-RGD in a preclinical model using patient-derived xenografts from subjects with advanced NSCLC (13), showing superior theranostic properties compared with non–EB-bound RGD. Another preliminary clinical study found that utilizing ^177^Lu-labeled dimeric RGD peptide to treat patients with TC resulted in improved biodistribution and prolonged retention in locoregional/metastatic lesions (35). Another avenue to extend the biological half-life of RGD analogs is to modify them using lipid particles. Addition of lipids like polyethylene glycol (PEG) into RGD peptides results in lower immunogenicity, as the peptide is protected from the rapid clearance by the immune system (36–38). In addition, there are efforts to embed RGD analogs into the liposomes. In order to decrease the risk of severe allergic response to liposomes, some studies have suggested that the introduction of an SGS (serine-glycine-serine) amino acid sequence in c(RGDyK)-modified liposomes may lead to reduced anaphylaxis events and improve the safety profile of drug delivery (39).

One of the strengths of our study is the utilization of the robust clinical data to evaluate the expression of α_V_β_3_ integrins in different types of TC. Moreover, our in vivo studies used new advances in RGD analog synthesis using stable cyclic analogs either in dimeric or monomeric forms and extending their biological stability by adding the EB moiety.

One of the limitations of our study is that the mechanisms of α_V_β_3_ expression, dimerization, and cell membrane trafficking in TC have not been investigated. However, we analyzed transcriptome and protein expression of the resected tumors and demonstrated that ^177^Lu-DOTA-EB-RGD induces a type I interferon response program that needs to be validated in immunocompetent model. In conclusion, the radiolabeled α_V_β_3_ analog ^177^Lu-DOTA-EB-RGD has potent growth inhibitory effects in TC characterized by a high α_V_β_3_ integrin expression that has been observed mostly in PTC. The α_V_β_3_ integrin could potentially serve as a molecular target for therapy with radiolabeled RGD analogs in a subset of TC that are characterized by a high ^68^Ga-DOTA-RGD_2_ uptake. Because kidneys appear to be dose-limiting organ, clinical translation should be preceded by pharmacokinetic studies that evaluate the addition of nephroprotective agents to PRRT.

## Methods

### Sex as a biological variable.

Our study examined male and female mice, and similar findings are reported for both sexes.

### Expression profiling of integrin α_V_β_3_.

Integrin α_V_β_3_ mRNA and/or protein expression was evaluated in 496 TCs included in TCGA, commercially available Human TC Tissue Microarray (TMA) slides (US Biomax Inc.), including 70 TC and 10 normal thyroid samples, and 14 TC cell lines.

### BRS.

*BRAF*-like or *RAS*-like expression profiles were determined through standard BRS scores ranging from –1 to 0 for *BRAF*-like and 0 to 1 for *RAS*-like TC (40). The association between BRS score and α_V_β_3_ expression was tested by Spearman’s correlation (*r*).

### Immunohistochemistry.

Immunostaining was performed on commercially available human TC tissue microarray slides (US Biomax Inc.). Sections were dewaxed, soaked in alcohol (descending alcohol series 100%, 96%, 70%), washed in deionized water, and then microwave-treated in antigen unmasking solution (Vector Laboratories) for 10 minutes. Endogenous peroxidase activity was quenched by incubation in BLOXALL Blocking Solution (Vector Laboratories) for 10 minutes. Sections were then incubated for 20 minutes in 2.5% horse serum and then at 4°C overnight with anti–integrin α_V_β_3_ antibody (clone EM22703, ZooMAb Rabbit Monoclonal, Sigma-Aldrich; diluted 1:100).

Immunostaining was performed using ImmPRESS Horse Anti-Rabbit IgG PLUS Polymer Kit (Vector Laboratories) as per the manufacturer’s instructions. Peroxidase staining was revealed with the ImmPact DAB peroxidase substrate kit (Vector Laboratories). Sections were counterstained with hematoxylin and mounted. The antiserum was omitted in the negative control.

The results of staining were interpreted and scored independently by 2 investigators. The evaluation of immunoreactivity scores (IHS) was carried out using ×200 magnification. Staining intensity (SI) was assessed according to a categorical scale: 0, no staining; 1, faint staining; 2, slight staining; 3, moderate staining; and 4, strong staining. The percentage of positively stained cells (PP) was assessed as the following: 0, no positive cells; 1, 0%–25% positive cells; 2, 26%–50% positive cells; 3, 51%–75% positive cells; and 4, 76%–100% positive cells. An overall IHS score was calculated by multiplying SI by PP (range of possible scores 0–16). The method for immunostaining TC tumor tissue microarrays derived from mouse xenografts used in this study is described in the Supplemental Methods.

### Cell culture.

Fourteen TC cell lines and 1 follicular thyroid cell line (HTori-3) were used for our study. These cell lines were either gifted by Electron Kebebew (Stanford University) or purchased from the University of Colorado Cancer Center, Sigma-Aldrich, or ATCC: FTC (FTC133); PTC (K1, BCPAP, and TPC1); medullary thyroid cancer (TT); anaplastic thyroid cancer (THJ16T, THJ29T, 8505C, OCUT2, KAT18, SW1736, and C643); and oncocytic thyroid cancer (XTC1). The cell lines were grown in the following media, based on recommended optimal conditions — HTori-3, FTC133, and K1: DMEM/F-12 (Gibco); XTC1, 8505C, OCUT2, and OCUT2-β3KO: DMEM (Gibco); TT: F12K (Gibco); TPC1, THJ16T, THJ29T, KAT-18, SW1736, BCPAP, and C643: RPMI1640 (Gibco); per the manufacturers’ instructions. All media were supplemented with 10% fetal bovine serum (R&D Systems), 10 U/mL penicillin-streptomycin (Gibco), and 0.25 μg/mL amphotericin B (Gibco). Luciferase-tagged FTC133 cell lines, designated as FTC133-Luc, were also maintained in respective media supplemented with G-418 (Geneticin) to preserve antibiotic selection of the luciferase lentivirus. The cells were authenticated by short tandem repeat analysis, and contamination was excluded via IMPACT II PCR analysis (Supporting Data Values).

### RT-PCR array.

For gene expression analysis, we used customized RT^2^ Profiler PCR Array plates manufactured by QIAGEN. The plate contained primers for 4 thyroid-specific genes: thyroid-stimulating hormone receptor (*TSHR*), sodium-iodine symporter (*NIS*), thyroglobulin (*TG*), thyroid peroxidase (*TPO*); 2 thyroid hormone receptor genes: thyroid hormone receptor α (*THRA*) and *THRB*; 18 integrin genes: integrin β1–β7 (*ITGB1*–*ITGB7*), integrin α1–α6 (*ITGA1*–*ITGA6*), integrin α_V_ (*ITGAV*), integrin α2B (*ITGA2B*), integrin αL (*ITGAL*), integrin αM (*ITGAM*), integrin αX (*ITGAX*); 5 housekeeping genes: β-actin (*ACTB*), glyceraldehyde-3-phosphate dehydrogenase (*GAPDH*), β-2-microglobulin (*B2M*), hypoxanthine phosphoribosyltransferase 1 (*HPRT1*), ribosomal protein lateral stalk subunit P0 (*RPLP0*); 1 human genomic DNA contamination control (*HGDC*); 1 positive PCR control (*PPC*); and 1 reverse transcriptase control (*RTC*). The gene expression within the cDNA samples was analyzed using iQ SYBR Green Supermix (Bio-Rad) on a StepOnePlus Real-Time PCR Detection System (Applied Biosystems). The relative gene expression was normalized to the housekeeping genes and calculated using the 2^–ΔΔCt^ method.

### Immunoblotting.

TC cells were lysed in M-PER (Mammalian Protein Extraction Reagent, Thermo Fisher Scientific) according to the manufacturer’s instructions. The protein concentration was calculated through the Bradford assay (DC protein assay Kit II, 5000112, Bio-Rad). The protein samples were loaded on 4%–12% SDS-PAGE gels (Novex; 40 μg/lane) and transferred to polyvinylidene difluoride (PVDF) membranes through a Trans-blot Turbo system (from Bio-Rad). The membranes were then blocked with 3% BSA diluted in PBST at room temperature for 1 hour. Next, the membranes were incubated at 4°C overnight with primary antibodies against α_V_β_3_ dimers (1:1000; Novus Biologics, SC56-07), α_V_ monomer (1:1000; Abcam, ab179475), β_3_ monomer (1:1000; Abcam, ab119992), or GAPDH (1:1,000; Invitrogen, MA5-15738) as housekeeping protein. The membranes were washed thrice with PBST and incubated further with either an anti-rabbit (1:1,000; sc-2357) or anti-mouse (1:1,000; sc-516102) secondary antibody (Santa Cruz Biotechnology) at room temperature for 1 hour. The membranes were then washed with PBST thrice and visualized through an Imaging System (Bio-Rad) using ECL (obtained from Azure Biosystems).

### Generation of integrin β3–knockout cell lines (ITGB3^–^/^–^) using CRISPR/Cas9 genome editing.

Guide RNAs (gRNA) targeting exon 3 (3′-GCGAGGUGAGCCCAGAGGCA-5′, 3′-UCACAGCGAGGUGAGCCCAG-5′, and 3′-UUCUCCUUCAGGUCACAGCG-5′) of the integrin β3 gene (*ITGB3*) were designed and synthesized by Synthego. Cas9 ribonucleoprotein (RNP) was synthesized by the UC Berkeley QB3 MacroLab. The Lipofectamine CRISPRMAX Cas9 Transfection Reagent (Thermo Fisher Scientific) was used to transfect the Cas9/gRNA complex into cells, following the manufacturer’s protocol. A 2:1 molar ratio of RNA/Cas9 was determined to be optimal. Cells were seeded 24 hours prior and transfected at 50% confluence in 24-well plates. Single cells were isolated using limiting dilutions and propagated to obtain single-cell–derived colonies. Mutations in the targeted region were determined by PCR amplification and capillary DNA sequencing. Knockout of the *ITGB3* gene was confirmed at the protein level by flow cytometry.

For capillary DNA sequencing, DNA was isolated from cells using the QIAGEN DNeasy Blood and Tissue Kit, and the target region amplified by PCR with the following primer pairs: 5′-GCTGGTCTACCAGGCATCTT-3′ and 3′-CCATTCTCTTGCCCACACCT-5′; and 5′-TCAGCTTCGGTTCCAAGGAC-3′ and 3′TGGATGACGGTGTCCAGTTG-5′, using the PCR Master Mix (Thermo Fisher Scientific) following the manufacturer’s instructions. The PCR product was cleaned using the Zymo Research DNA Clean & Concentrator kit and sequenced by capillary DNA sequencing. Raw sequences were analyzed using Chromas DNA sequence analysis software (Technelysium, https://technelysium.com.au/) version 2.6.6. The alignment programs EMBOSS WATER Pairwise Sequence Alignment (EMBOSS WATER < Job Dispatcher < EMBL-EBI) and Clustal Omega Multiple Sequence Alignment (Clustal Omega < Job Dispatcher < EMBL-EBI) were employed for pairwise and multiple sequence alignments respectively.

### Adenovirus for hITGAV and hITGB3 and α_V_β_3_ overexpression.

Adenoviruses expressing human *ITGAV* (NCBI: NM_002210.5) and *ITGB3* (NCBI: NM_000212.3), genes corresponding to α_V_ and β_3_ proteins, were custom-designed (Supplemental Figure 2) and procured from VectorBuilder Inc. After titration to determine transfection efficiency, 10 MOI/cell for each adenovirus was used throughout the study. Cells (2 × 10^5^) were seeded overnight and treated with desired concentration of adenovirus for 48 hours in fresh media, then harvested to isolate total RNA according to the RNeasy Mini Kit (QIAGEN, 74106). cDNA was prepared using High-Capacity cDNA Reverse Transcription Kit (Applied Biosystems, 4368813), and real-time qPCR was performed to determine overexpressed genes using the TaqMan Gene Expression Assay for h*ITGAV* (Hs00233808_m1) and h*ITGB3* (Hs01001469_m1). After confirming satisfactory overexpression efficiency and time point, α_V_β_3_ dimer protein levels were analyzed.

### Cell surface staining of α_V_β_3_.

FTC133 and OCUT2 cells were analyzed for live-cell surface expression of α_V_β_3_ using flow cytometry. Optimally maintained cells were detached with Accutase cell detachment solution (Innovative Cell Technologies, AT-104), washed with PBS followed by FACS buffer (PBS, 5% FBS, 0.1% NaN_3_), and 2 × 10^5^ cells were used in each sample for staining. Rabbit anti-α_V_β_3_ monoclonal antibody (1:250; Novus Biologicals, NBP2-67557) was added to designated samples and incubated for 90 minutes. Alexa Fluor 633–conjugated (AF633-conjugated) F(ab′)_2_-Goat anti-Rabbit IgG (H+L) (Thermo Fisher Scientific, A-21072) secondary antibody was added at a dilution of 1:1000 after the primary antibody incubation was complete. Finally, DAPI was added as a viability marker, and flow cytometric analysis was performed with 10,000 events counted for each sample. The blank control contained only secondary antibody and DAPI. The flow cytometry results were analyzed using FlowJo v10 software (https://www.flowjo.com/flowjo10/overview), and the built-in geometric mean analysis was employed to calculate the MFI. MFI measures the fluorescence intensity of a specific cell subpopulation, normalized to cell number, to quantify that subpopulation’s contribution to the overall marker expression in the entire cell population (41).

For the comparison of the cell surface expression of α_V_β_3_ receptor in parental OCUT2 and CRISPR/Cas9 *ITGB3*-knockout OCUT2 (named OCUT2-β3KO) cell lines, cells were prepared as described above and stained with mouse anti–integrin α_V_β_3_ monoclonal antibody (1:200; Abcam, ab7166), followed by AF647-conjugated Goat anti-Mouse IgG (H+L) Cross-Adsorbed secondary antibody (Thermo Fisher Scientific, A-21235) added at a dilution of 1:1000. For the parental FTC133 cells and FTC133 cells with adenoviruses overexpressing *ITGAV* and *ITGB3* (named FTC133-α_V_β_3_^+^), cell surface α_V_β_3_ dimer staining was done in the same way after adenoviral transfection was completed.

### Synthesis of radiolabeled peptides.

The radiolabeled peptides ^68^Ga-DOTA-c(RGDfK)_2_, a cyclic RGD dimer labeled with ^68^Ga via DOTA as a linker (named ^68^Ga-DOTA-RGD_2_), and ^177^Lu-DOTA-EB-cRGDfK, a cyclic RGD labeled with ^177^Lu via DOTA as a linker and attached to Evans Blue (EB) moiety (alias ^177^Lu-DOTA-EB-RGD), were synthesized following a previously described method (13, 15). Briefly, 500 μL of ^68^Ga elution fraction (obtained from the iThemba, South Africa, generator) in 0.6 M HCl was added to a solution of 100 μL 5 M NaOH and 500 μL 1 M HEPES (pH 7.2). This solution was then added to 20 μg (1 mg/mL in water) of in-house synthesized DOTA-c(RGDfK)_2_. ^68^Ga-DOTA-c(RGDfK)_2_ was obtained by incubating the reaction mixture at 85°C for 10 minutes. The reaction was diluted into 5 mL of water and passed through BondElute (C-18 100 mg, Agilent). The cartridge was washed with 10 mL of water, and the product was eluted twice with 150 μL 80% ethanol containing 1 mM HCl. The column-purified radiotracer was diluted in PBS for administration in mice. Radiochemical purity was greater than 95 %, as assayed by radio HPLC.

The 30 μL stock solution of 1.94 GBq ^177^LuCl_3_ was diluted with 0.25 mL 0.4 M sodium acetate (pH 5) and mixed with 50 μg (1 mg/mL) in-house synthesized DOTA-EB-cRGDfK (33). After a 20-minute incubation at 95°C on an orbital shaker, the ^177^Lu-DOTA-EB-cRGDfK reaction solution was assayed by rapid TLC on iTLC (silica on fiber glass) with 0.1 M citric acid, pH 5. The iTLC plate was assayed with a Bioscan TLC plate reader. Radiochemical purity was greater than 90%, and the reaction solution was used directly following dilution with PBS prior to injection.

### Determination of maximum binding sites (B_max_) and binding affinity (K_d_).

Saturation binding analysis was performed to determine the α_V_β_3_ receptor densities and radiopeptide binding affinity of cell lines (42). Parental OCUT2, OCUT2-β3KO, FTC133, and FTC133-α_V_β_3_^+^ cell lines were cultured in their respective media and maintained at 60%–70% confluence. Cells were individually seeded at a density of 4–5 × 10^4^ per well in 48-well flat-bottom microplates and recuperated overnight at 37°C. The wells were designated as non-specific binding (NSB) and total binding (TB). For the FTC133-α_V_β_3_^+^ cell line, cells were counted after adenoviral transfection was complete and before NSB or TB designation. Increasing concentrations of ^68^Ga-labeled peptide, ^68^Ga-DOTA-RGD_2_ (10–3000 nM), were added, and cells were incubated at 37°C for 60 minutes. Cold (^68^Ga-unlabeled) peptide at a 5× concentration relative to the highest radiolabeled peptide concentration (i.e., 15 μM) was used as a blocking solution to pretreat cells in the NSB wells. Cells were washed, the bound fractions were collected using 1.5 M NaOH, and the radioactivity was counted with a well-type automatic γ counter (2480 Wizard2, PerkinElmer). Specific binding counts were obtained by subtracting NSB counts from TB counts and converted to specifically bound radioligand (in fmol). Nonlinear regression analysis with extra-sum-of-squares *F* test was used to analyze data from at least 3 different experiments and calculate K_d_ and B_max_ values using the 1-site specific binding function (*Y* = B_max_ × *X*/[K_d_ + *X*], where *X* = radioligand concentration and *Y* = specific binding) in GraphPad Prism (43), and Welch’s *t* test was used to compare the B_max_ and K_d_ values.

### Animal studies.

Mice were housed up to 5 per cage**,** provided ad libitum access to water and standard chow (NIH-31 formula; 5L7X, Animal Specialties and Provisions). The animal room was maintained at 22°C with 50% humidity and a 12:12-hour light/dark cycle with lights off at 6 pm. Experiments were performed with equal numbers of male and female mice ranging from 8 to 13 weeks of age, with a starting weight of 20 g or more. To generate cell-derived xenografts (CDXs), athymic nude mice (NU/J, strain 002019, The Jackson Laboratory) were injected subcutaneously with 5 × 10^6^ OCUT2 cells. Tumor growth and mouse weights were measured at regular intervals, and treatment was started when the average tumor size reached approximately 200 mm^3^. Along with the consideration of similar average tumor size within each group, mice were randomly assigned to maintain an equal sex ratio and body weight across groups prior to treatment initiation. The treatment groups were as follows: (a) placebo group *n* = 8 mice: PBS via oral gavage daily and via tail vein injection once; (b) lenvatinib group *n* = 8: lenvatinib 10 mg/kg via daily oral gavage and PBS via tail vein injection once; (c) ^177^Lu group *n* = 8: 0.5 mCi of ^177^Lu-DOTA-EB-RGD via tail vein injection and PBS via oral gavage daily; (d) ^177^Lu+L group *n* = 8: 0.5 mCi of ^177^Lu-DOTA-EB-RGD via tail vein injection and lenvatinib 10 mg/kg via daily oral gavage. 200 μL was the delivered volume for all treatments.

Static PET imaging for ^68^Ga-DOTA-RGD_2_ (100 μCi) was conducted 60 minutes after injection. An Inveon scanner (Siemens) was utilized to capture PET images. Thereafter, the images were analyzed using ASIPro software (Siemens) to determine regions of interest and quantify SUVs. The SUVs were further corrected with respect to the injected radioactivity dose, decay values, branching, and respective mouse weight. After the last scan, mice were euthanized and tumors, heart, liver, and kidneys were collected to perform biodistribution studies. Ex vivo quantification of relative γ radioactivity in tumors and normal organs (liver, heart, and kidneys) was performed by direct measurement using a γ counter (2480 Wizard2, PerkinElmer) 1 hour after the conclusion of the experiment. The counts per minute (CPM) readings were normalized by the weight of the tissues exposed to ^177^Lu that was received by 2 groups of animals (^177^Lu and ^177^Lu+L) 16 days before the conclusion of the experiment and are represented as relative values for each group.

For the generation of metastatic nodules of TC in the lungs, extremely immunodeficient mice, NOD.Cg-*Prkdc^scid^* *Il2rg^tm1Wjl^*/SzJ, commonly referred to as NSG mice, were procured from The Jackson Laboratory (strain 005557). FTC133-Luc cells (5 × 10^5^) were injected i.v. through the tail vein to generate metastatic nodules in the lungs. The presence of lung and/or bone metastases was monitored weekly by bioluminescence quantification via IVIS imaging (IVIS Lumina Series III, Revvity). Mice were intraperitoneally injected with a freshly prepared solution of D-luciferin (potassium salt, Regis Technologies, 1-360222-200, 150 mg/kg body weight) in sterile PBS and anesthetized by isoflurane inhalation before IVIS imaging was performed with automatic acquisition and luminescent exposure of 60 seconds. Regions of interest (ROIs) of the same area were marked in the captured images of the animals, and the built-in analysis function was used to determine the levels of bioluminescence in terms of total counts. IVIS imaging was performed on the days of inoculation, start of treatment, and euthanasia. Sex-unbiased randomly divided mice with similar average tumor size and/or metastatic tumor burden quantified by IVIS bioluminescence with a threshold of >10^4^ counts, were categorized into 4 groups (*n* ≥ 6): (a) placebo, designated P, received vehicle only; (b) lenvatinib alone (L) received 10 mg/kg body weight lenvatinib via oral gavage; (c) ^177^Lu-DOTA-EB-RGD (^177^Lu) subjected to monotherapy with 0.5 mCi radiotherapy tracer DOTA-EB-RGD conjugated to ^177^Lu (*t*_½_ 6.6 days) via tail vein injection, or (d) in combination with 10 mg/kg body weight lenvatinib (^177^Lu+L). All the treatment groups received placebo doses complementary to other groups via i.v. or p.o. at the same rate to normalize the levels of stress in animals among groups.

### RNA-seq analysis of resected tumors.

Total RNA from each tumor was quantified through a NanoDrop ND-1000. The library preparation was done using 1–2 g of the total RNA. Thereafter, an Agilent 2100 Bioanalyzer was employed to verify the completed libraries and quantified through the method of absolute quantification qPCR. Next, the barcoded libraries were mixed and further denatured in NaOH to single-stranded DNA, recorded on an Illumina flow cell, then amplified in situ, followed by sequencing with an Illumina NovaSeq 6000 instrument for 150 cycles.

The quality of the sequence was assessed using the FastQC software version 0.11.7 (https://www.bioinformatics.babraham.ac.uk/projects/fastqc/). The trimmed reads (trimmed 5′, 3′-adaptor bases utilizing cutadapt 1.17 (https://cutadapt.readthedocs.io/en/stable/) were then aligned to the reference genome with the Hisat2 software version 2.1.0 (https://cutadapt.readthedocs.io/en/stable/). StringTie 1.3.3 (https://github.com/gpertea/stringtie) was used to analyze the transcript abundances in each sample and the R package Ballgown 2.10.0 (https://bioconductor.org/packages/3.22/bioc/html/ballgown.html) was used to calculate the FPKM (fragments per kilobase of exon per million mapped reads) value for gene and transcript level. Thereafter, the R package Ballgown was used in order to filter the differentially expressed transcripts and genes. Next, the assembled results were employed to predict the new genes and transcripts through comparison with the reference annotation (by StringTie and Ballgown). Furthermore, detection of alternative splicing plot and events was performed through Replicate Multivariate Analysis of Transcript Splicing (rMATS). Correlation analysis and Principal Component Analysis (PCA) were based on FPKM. Gene Ontology, Hierarchical Clustering, gene expression level, Pathway analysis, volcano plots and scatter plots were conducted using the differentially expressed genes in Python 2.7 (https://www.python.org/), R or shell environments for the graphics as well as statistical computing. The RNA-seq was performed by Arraystar Inc.

### Statistics.

The normal distribution of continuous data is represented as mean with standard deviation (mean ± SD) or standard error of mean (mean ± SEM), and parametric tests were used for intergroup comparisons (*t* test or ANOVA for more than 2 groups) as appropriate. For non-normally distributed data, non-parametric tests such as the Mann-Whitney *U* test or the Wilcoxon rank-sum test were used. Mixed-effects model was used to assess the differences in the tumor growth rates in the 4 groups of mice subjected to different treatment modalities, and non-linear regression modeling was used for binding assays. GraphPad Prism 10.2.3 software was utilized to analyze the data, and a *P* value of 0.05 or less was considered to be statistically significant.

### Study approval.

All animals were maintained at NIH facilities in accordance with the guidelines established by the Association for Assessment and Accreditation of Laboratory Animal Care (AALAC), and all protocols were approved by the Institutional Animal Care and Use Committee (protocol: K122-MDB-24) at the National Institute of Diabetes and Digestive and Kidney Diseases (NIDDK).

### Data availability.

Raw data for all the experiments performed in this study as well as next-generation sequencing data are stored in the publicly available NCBI Gene Expression Omnibus (accession number GSE343650). The analytical codes are submitted in Github (commit URL: https://github.com/sonamkumari2025/RNAseq-Volcano-Plot.git; and the ID: 8a88f17ae69d2c24031898a21fba92b8fcde78d7). All the raw data from which the plots are generated in the main and supplemental figures are submitted as a Supporting Data Values file.

### Statement on use of AI.

On June 3, 2026, ChatGPT v.5.5, US Department of Health Enterprise was used to conceptualize the graphical abstract and was created using BioRender. Prompt used: “Using the results summary create graphical abstract. Do not recapitulate the data, rather put schematic of take-home message.”

## Author contributions

SK: conception, design, data acquisition, preparation of figures, analysis and interpretation, writing the manuscript, critical review and editing the manuscript. RS: conception, design, data acquisition, preparation of figures, analysis and interpretation, writing the manuscript, critical review and editing the manuscript. ZW: data acquisition, critical review and editing the manuscript. ST: data analysis and interpretation, critical review and editing the manuscript. LA: data acquisition, critical review and editing the manuscript. OG: data acquisition, critical review and editing the manuscript. HL: data acquisition, critical review and editing the manuscript. NB: data analysis, critical review and editing the manuscript. LL: data acquisition, critical review and editing the manuscript. DK: data acquisition, critical review and editing the manuscript. VV: data acquisition, critical review and editing the manuscript. JKG: conception, design, data interpretation, writing the manuscript, critical review and editing and finalizing the manuscript, overall supervision. VV and JKG: interpretation and scoring of immunostaining results. All authors took part in reviewing the manuscript and revising its intellectual and technical content. All authors gave approval of the version to be published and agreement to be accountable for all aspects of the work.

## Conflict of interest

The authors have declared that no conflict of interest exists.

## Funding support

This work is the result of NIH funding, in whole or in part, and is subject to the NIH Public Access Policy. Through acceptance of this federal funding, the NIH has been given a right to make the work publicly available in PubMed Central.

- NIDDK Intramural Program ZIE DK 04705313.

## Supplementary Material

Supplemental data

Unedited blot and gel images

Supporting data values

## Figures and Tables

**Figure 1:**
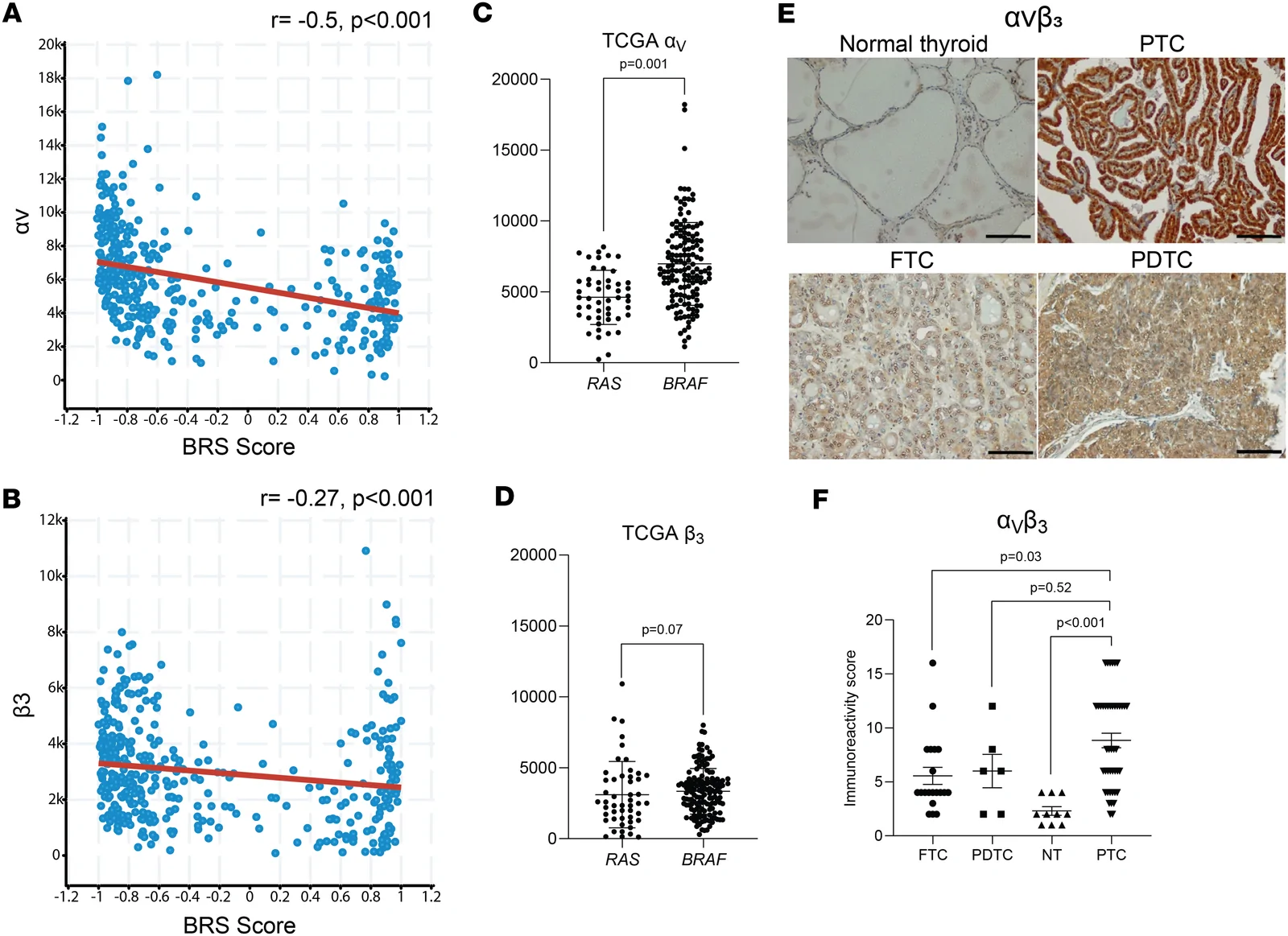
Expression of α_V_β_3_ integrins. (**A**) A moderate negative correlation (Spearman, 2-sided) between mRNA expression of α_V_ and BRS (*r* = –0.5, *P* < 0.001) was observed. (**B**) A weak negative correlation (Spearman, 2-sided) between mRNA expression of β_3_ and BRS (*r* = –0.27, *P* < 0.001) (*n* = 496). (**C**) The Cancer Genome Atlas (TCGA) was utilized to evaluate the RNA-seq data in selected samples with *BRAFV600E* mutation (*n* = 143) and *HRAS/NRAS/KRAS* pathogenic variants (*n* = 49) and observed that α_V_ expression was significantly higher (1-tailed non-parametric *t* test, Mann-Whitney, *P* = 0.001) in *BRAFV600E*-driven TC in comparison to *RAS*-driven tumors. (**D**) Non-significant (1-tailed non-parametric *t* test, Mann-Whitney) difference in β_3_ mRNA expression in *BRAFV600E* (*n* = 143) vs. *RAS*-mutant tumors (*n* = 49). (**E**) Representative immunostaining revealing a higher α_V_β_3_ dimers expression in papillary TC (PTC) compared with follicular TC (FTC) (*P* = 0.03), and normal thyroid (*P* < 0.001). Original magnification, ×100. Scale bars: 100 μm. (**F**) Quantitative analysis of the immunostaining of α_V_β_3_ dimers expression in different thyroid tissues (1-way ANOVA, Kruskal-Wallis). Normal thyroid (NT) *n* = 10, PTC *n* = 44, FTC *n* = 20, PDTC *n* = 6.

**Figure 2:**
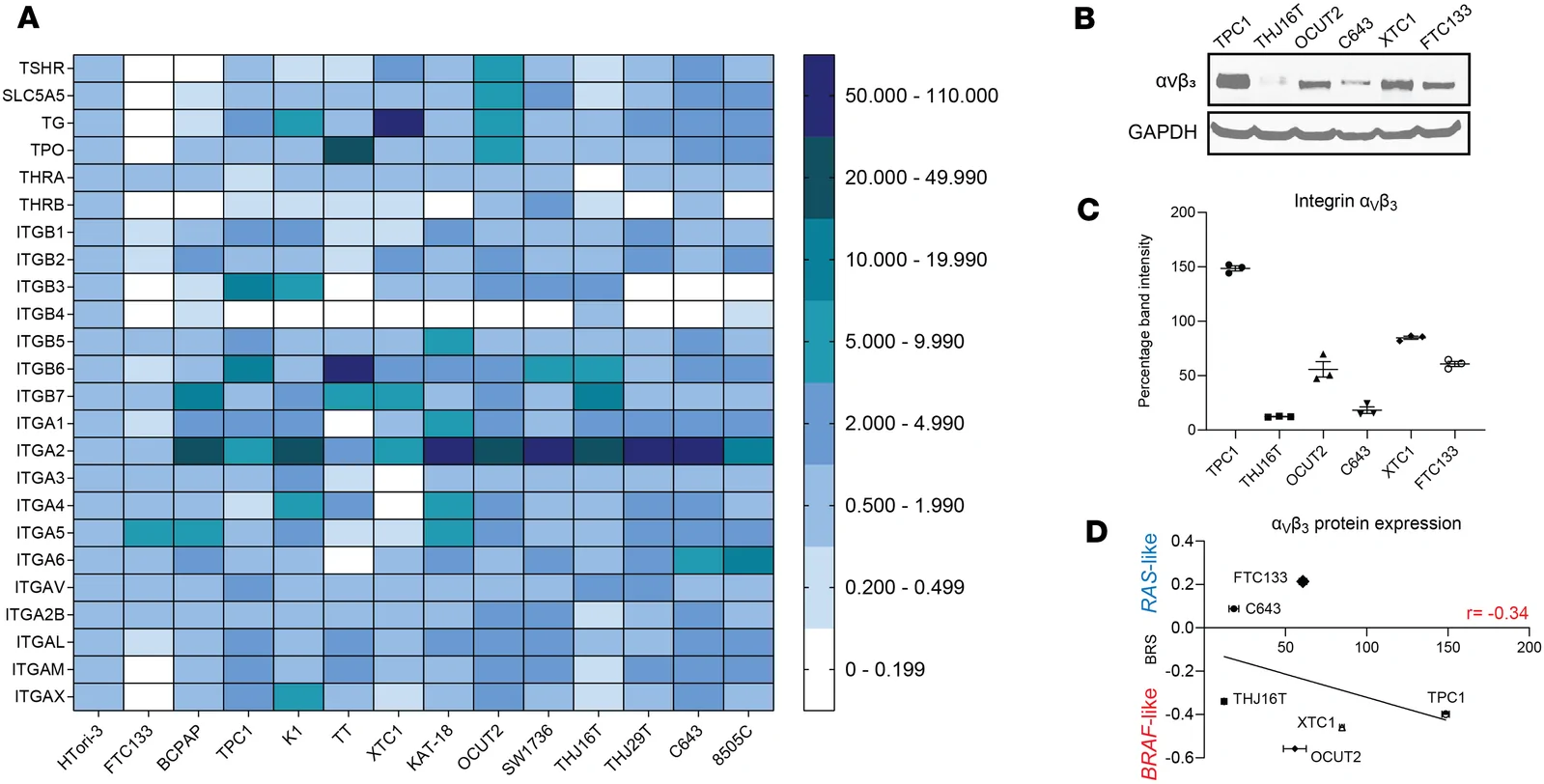
Comparison of α_V_β_3_ expression. (**A**) Relative mRNA expression analysis of thyroid-specific genes, thyroid hormone receptor genes, and integrins in 13 TC cell lines in comparison to a normal thyrocyte cell line, HTori-3 (*n* = 3). TC cell lines are characterized by variable expression of *ITGAV* (α_V_) and *ITGB3* (β_3_). (**B**) Immunoblot depicting the expression of α_V_β_3_ dimers in different TC cell lines. The highest expression was found in TPC1 (*CCDC6-RET* fusion), followed by oncocytic TC cell line XTC1, OCUT2 (*BRAF*-like TC), and FTC133 (*RAS*-like TC). C643 (with *HRAS p.G13R* mutation) and THJ16T (with *MKRN1-BRAF* fusion) cell lines had very minimal expression of α_V_β_3_ protein. (**C**) Quantification of the Western blot demonstrating α_V_β_3_ dimer protein expression (*n* = 3). (**D**) Low non-significant correlation (Pearson, 2-tailed) between α_V_β_3_ protein expression normalized to *GAPDH* (*x*-axis) and *BRAF/RAS* transcriptome profile quantified as *BRAF-RAS* score (BRS, *y*-axis): *r* = –0.34 (*n* = 3).

**Figure 3:**
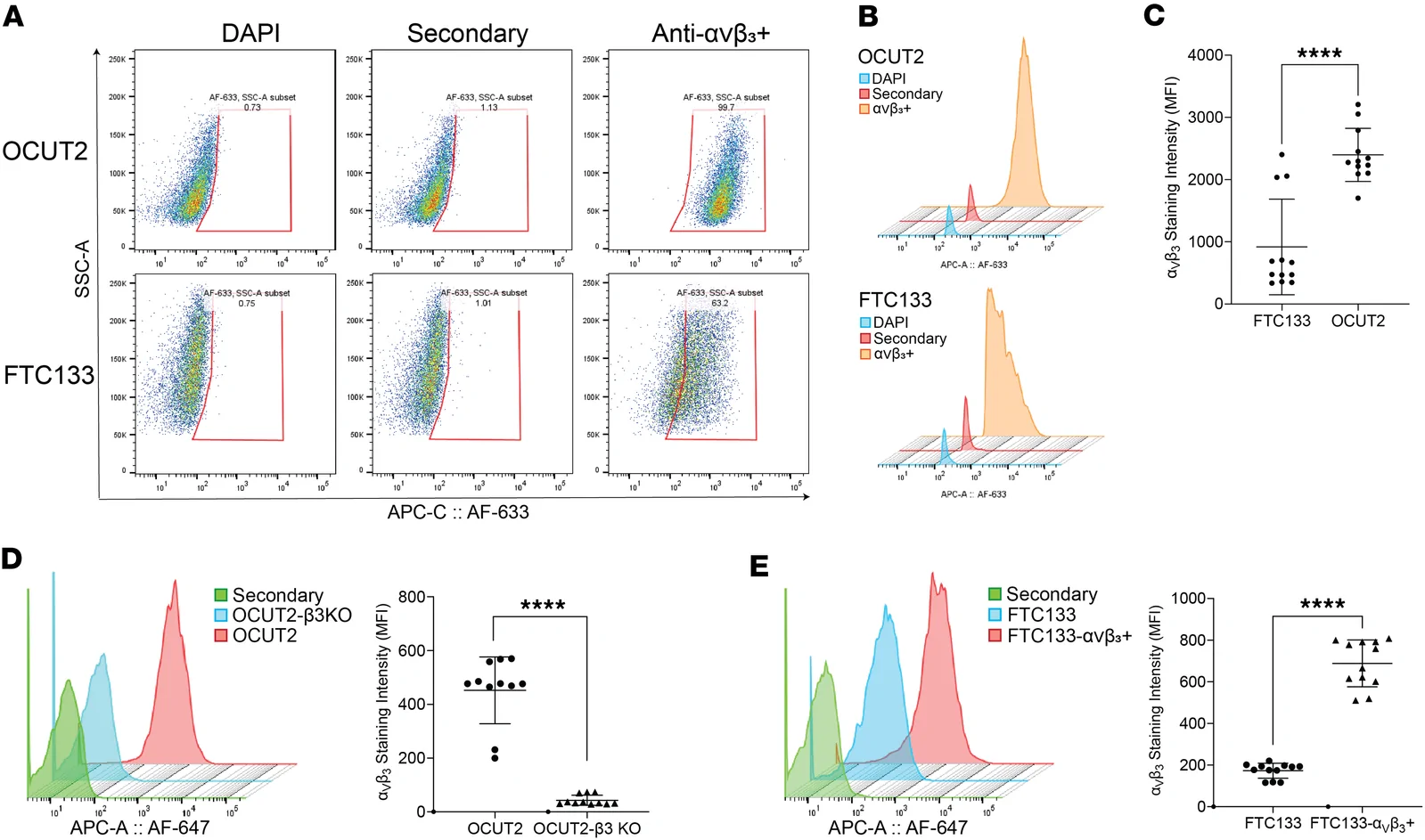
Evaluation of α_V_β_3_ integrin receptor on TC cell surface. (**A**) Representative flow cytometry dot plots (10,000 events acquired; *N* = 3, *n* = 3) showing cell-surface expression of integrin α_V_β_3_ in OCUT2 (99% positive, upper panel) and FTC133 (63% positive, lower panel) cells. Red gates indicate α_V_β_3_-positive cells detected by AF633-conjugated secondary antibody fluorescence (APC channel, *x*-axis) versus side scatter (*y*-axis). Plots are shown for DAPI, secondary antibody control, and anti-α_V_β_3_–stained cells. (**B**) Overlay histograms of α_V_β_3_ staining show a greater rightward fluorescence shift in OCUT2 cells compared with FTC133 cells, indicating higher α_V_β_3_ expression. (**C**) The mean fluorescence intensity (MFI) analysis revealed higher α_V_β_3_ cell surface expression in OCUT2 than in FTC133. The results show an average of *N* = 4 biological replicate experiments, each done in triplicate (*n* = 3) and 2-tailed Welch’s *t* test was performed. (**D**) The staggered overlay for the univariate histograms of α_V_β_3_ staining for OCUT2-β3KO (blue) over OCUT2 parental cells with secondary (green) and anti-α_V_β_3_ (red) antibodies shows no expression of α_V_β_3_ receptor on the knockout cell surface. The adjacent dot plot quantitatively reveals the significant downregulation in MFI (*N* = 4, *n* = 2 or 3, 2-tailed Welch’s *t* test). (**E**) Adenoviral overexpression of α_V_β_3_ on FTC133 (red) resulted in a substantial shift of the staggered histogram compared with parental FTC133 (blue) and parental cells with secondary antibody (green). The corresponding MFI analysis showed a significant increase in α_V_β_3_ cell surface expression in the overexpressing cells compared with the parental cells (*N* = 4, *n* = 3, 2-tailed Welch’s *t* test). ****P < 0.0001.

**Figure 4:**
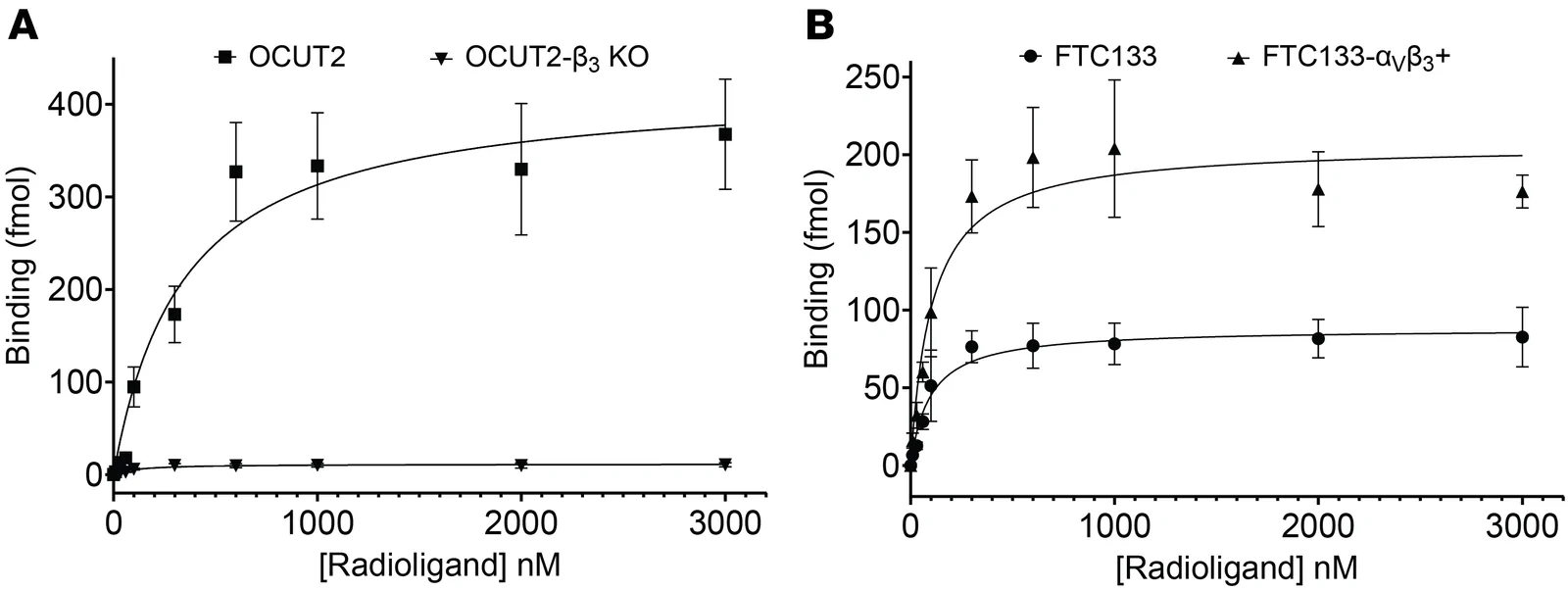
^68^Ga-DOTA-RGD_2_ saturation binding assay. The nonlinear regression curves showed saturation binding of the radioligand ^68^Ga-DOTA-RGD_2_ on (**A**) OCUT2 and OCUT-β3KO. OCUT-β3 KO display negligible binding compared with parental cells (mean ± SEM 11.3 ± 1.7, extra-sum-of-squares *F* test, *F*(2,176) = 907.8, *P* < 0.0001, *N* = 3, *n* = 3) (**B**) FTC133 and FTC133 α_V_β_3_-overexpressing cells (named FTC133-α_V_β_3_^+^). FTC133-α_V_β_3_^+^ have markedly high binding compared with parental FTC133 (mean ± SEM 206.6 ± 24.5, extra-sum-of-squares *F* test, *F*(2,116) = 206.6, *P* < 0.0001, *N* = 2, *n* = 3).

**Figure 5:**
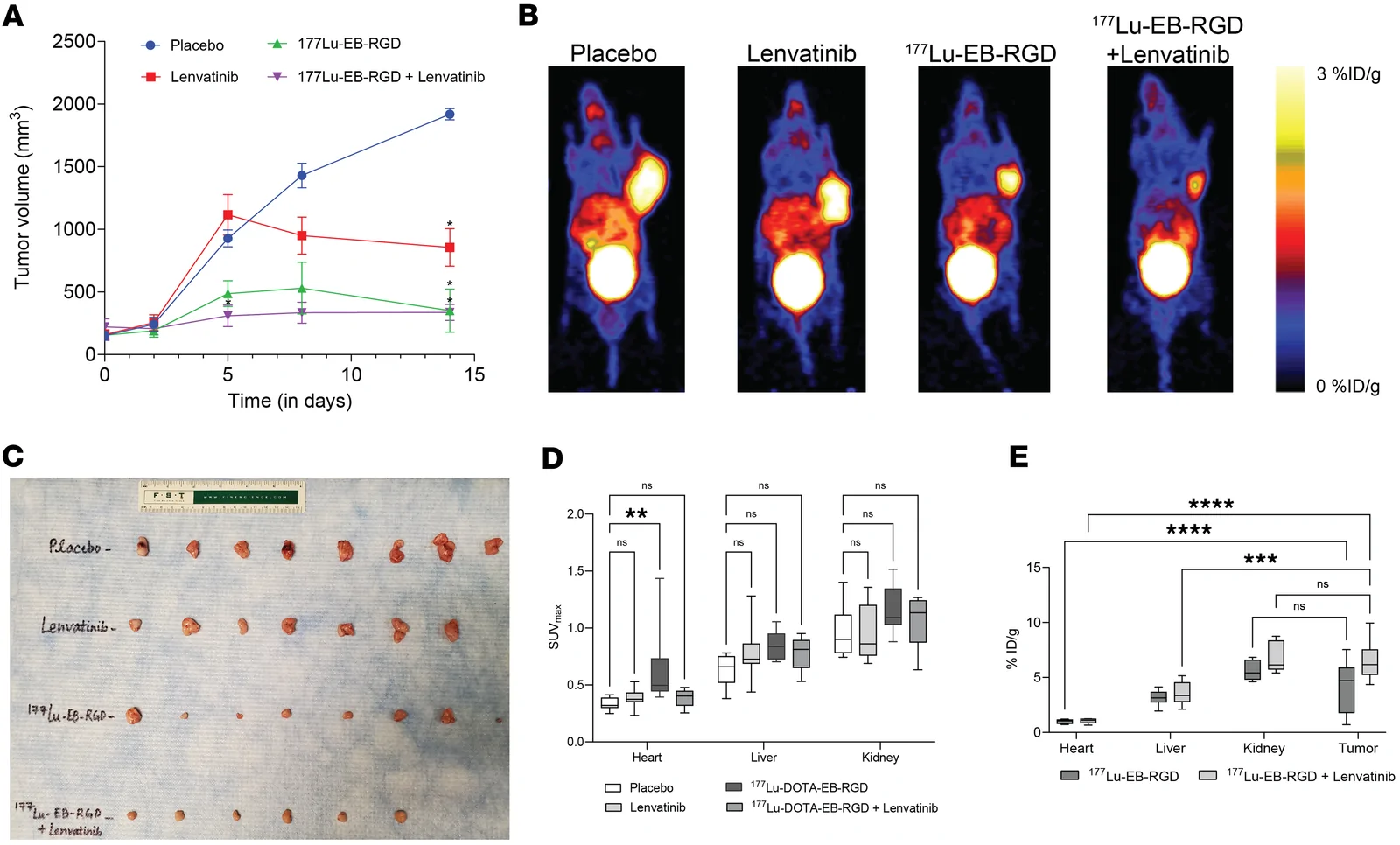
^177^Lu-DOTA-EB-RGD treatment in subcutaneous TC tumor models. (**A**) Tumor growth velocity analysis by 2-way mixed-effects model ANOVA revealed that treatment with ^177^Lu-DOTA-EB-RGD (^177^Lu) led to significant tumor growth reduction over time compared with placebo (*P* < 0.001) and lenvatinib alone (*P* < 0.001). Combination therapy (^177^Lu+L) decreased tumor growth compared with lenvatinib monotherapy (*P* < 0.001), although having similar effect as ^177^Lu monotherapy (*P* = 0.79). (**B**) Representative PET scan images depicting uptake of ^68^Ga-DOTA-RGD_2_ in tumors showing significantly lower uptake in mice exposed to combination therapy (^177^Lu+L, SUV_max_ 0.75 ± 0.11) in comparison with the placebo group (SUV_max_ 1.12 ± 0.11; *P* = 0.001) as well as lenvatinib (0.98 ± 0.21; *P* = 0.045) (1-way ANOVA with Holm-Šídák multiple-comparison test). (**C**) Resected tumors collected from each group show that treatment with ^177^Lu in monotherapy or combination therapy led to a significant reduction of tumor volume (median [IQR], 2-way mixed-effects model ANOVA) with respect to placebo [P] (^177^Lu 0.350 cm^3^ [0.172–0.508] vs. P 0.928 cm^3^ [0.196–1.674], *P* = 0.0001; and ^177^Lu+L 0.310 cm^3^ [0.214–0.335] vs. P 0.928 cm^3^ [0.196–1.674], *P* < 0.0001). The combination therapy (^177^Lu+L, 0.337 cm^3^) resulted in decreased tumor volume as compared with monotherapy with lenvatinib (0.855 cm^3^, *P* = 0.05) but had a similar effect as compared with ^177^Lu monotherapy (*P* = 0.99). (**D**) No treatment-associated difference in ^68^Ga-DOTA-RGD distribution at the conclusion of the experiment in normal organs other than from higher variability in heart exposure in ^177^Lu monotherapy as compared with placebo (2-way ANOVA). (**E**) Ex vivo biodistribution studies in ^177^Lu monotherapy and ^177^Lu +L revealed that the kidneys had a similar radiation exposure to the tumors, while exposure to the heart and liver was significantly lower (2-way ANOVA). The number of mice (*n*) studied for each group was: placebo, *n* = 8; lenvatinib, L, *n* = 7; ^177^Lu, *n* = 8; ^177^Lu+L, *n* = 6. **P < 0.01; ***P < 0.001; ****P < 0.0001.

**Figure 6:**
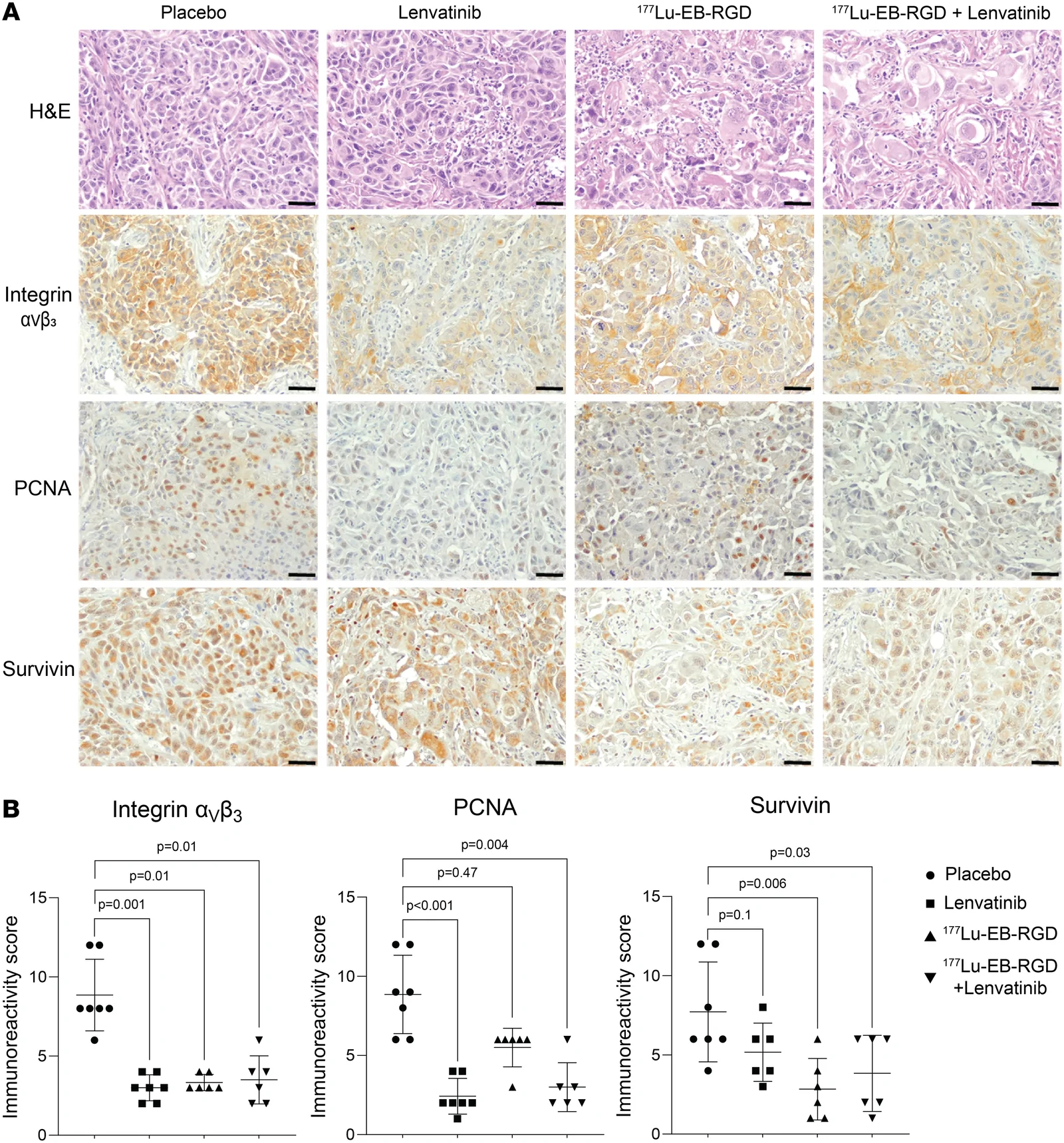
Immunohistochemistry of tumor tissues. (**A**) Representative images of immunostaining for α_V_β_3_ integrin, proliferation marker PCNA, and survival marker survivin (placebo *n* = 7, lenvatinib *n* = 7, ^177^Lu-DOTA-EB-RGD *n* = 6, ^177^Lu-DOTA-EB-RGD + lenvatinib *n* = 6). Original magnification, ×200. Scale bars: 100 μm. (**B**) Quantitative analysis of immunostaining of different groups with respect to control showing that compared to the tumors treated with placebo, the expression of integrin α_V_β_3_ dimers (1-way ANOVA, Kruskal-Wallis) was significantly reduced in lenvatinib (*P* = 0.001), ^177^Lu monotherapy group (*P* = 0.01), and ^177^Lu+L combination therapy (*P* = 0.01). Proliferation marker PCNA (1-way ANOVA, Kruskal-Wallis) was significantly reduced in the tumors exposed to combination therapy with ^177^Lu+L (*P* = 0.004) and monotherapy with lenvatinib with respect to placebo (*P* < 0.001). Also, in comparison with the tumors treated with placebo (Welch’s *t* test), survivin (cancer cells’ survival marker) was found to be significantly reduced in ^177^Lu+L combination therapy (*P* = 0.03) and ^177^Lu monotherapy (*P* = 0.006).

## Version 1 10/08/2026

Electronic publication
