# Supplementary material for: 177Lu-labeled DOTA-EB-RGD as a candidate for thyroid cancer therapy
Source: JCI Insight. 2026 Oct 8;11(19):e200594. doi: 10.1172/jci.insight.200594 (PMC13726428; doi:10.1172/jci.insight.200594)
Supplement: Supplementary file 1 [file jciinsight-11-200594-s027.pdf]

Supplemental Method: Immunohistochemistry

Single IHC staining performed on the Bond RX autostainer (Leica Biosystems) used the Bond Polymer Refine Kit (Leica Biosystems DS9800, with omission of the PostPrimary reagent) and the following conditions: Epitope Retrieval 1 (Citrate) for pan-AKT (Cell Signaling Technology #4691, 1:300, overnight 4°C), phospho-p44/42 (ERK1/2, Cell Signaling Technology #4376, 1:200, overnight 4°C), and BAX (Abcam #ab81083, 1:50, 30'); Epitope Retrieval 2 (EDTA) for PTEN (Abcam #ab267787, 1:2000 30'), phospho-MEK (Cell Signaling Technology #2338S, 1:50 60'), FAK (Abcam #ab40794, 1:250 30'), and BCL2 (Abcam #ab182858, 1:500 30'). Isotype control reagent was used in place of primary antibodies for the negative controls. Comparable positive staining was done in tissue samples characterized for the particular protein. Other reagents used were normal goat serum, biotinylated goat anti-rabbit IgG (Vector Labs), ABC-HRP (Vector Labs), and DAB. Slides were removed from the Bond autostainer, counterstained with hematoxylin, dehydrated through ethanols, cleared with xylenes, and coverslipped.

Slides were scanned at 40x using an Aperio AT2 scanner (Leica Biosystems) and analyzed with HALO (Indica Labs, v4.1). Staining quality and specificity were reviewed by a board-certified pathologist, and regions of interest were annotated to include tumor while excluding necrosis and slide-processing artifacts. The cytonuclear algorithm (Indica Labs, v2.0.9) was optimized to refine cell segmentation and staining thresholds. For quantitation, the percentage of positive cells or an H-score was calculated, where H-score = (1 × % weak staining) + (2 × % moderate staining) + (3 × % strong staining).

Supplemental Figure and Legends

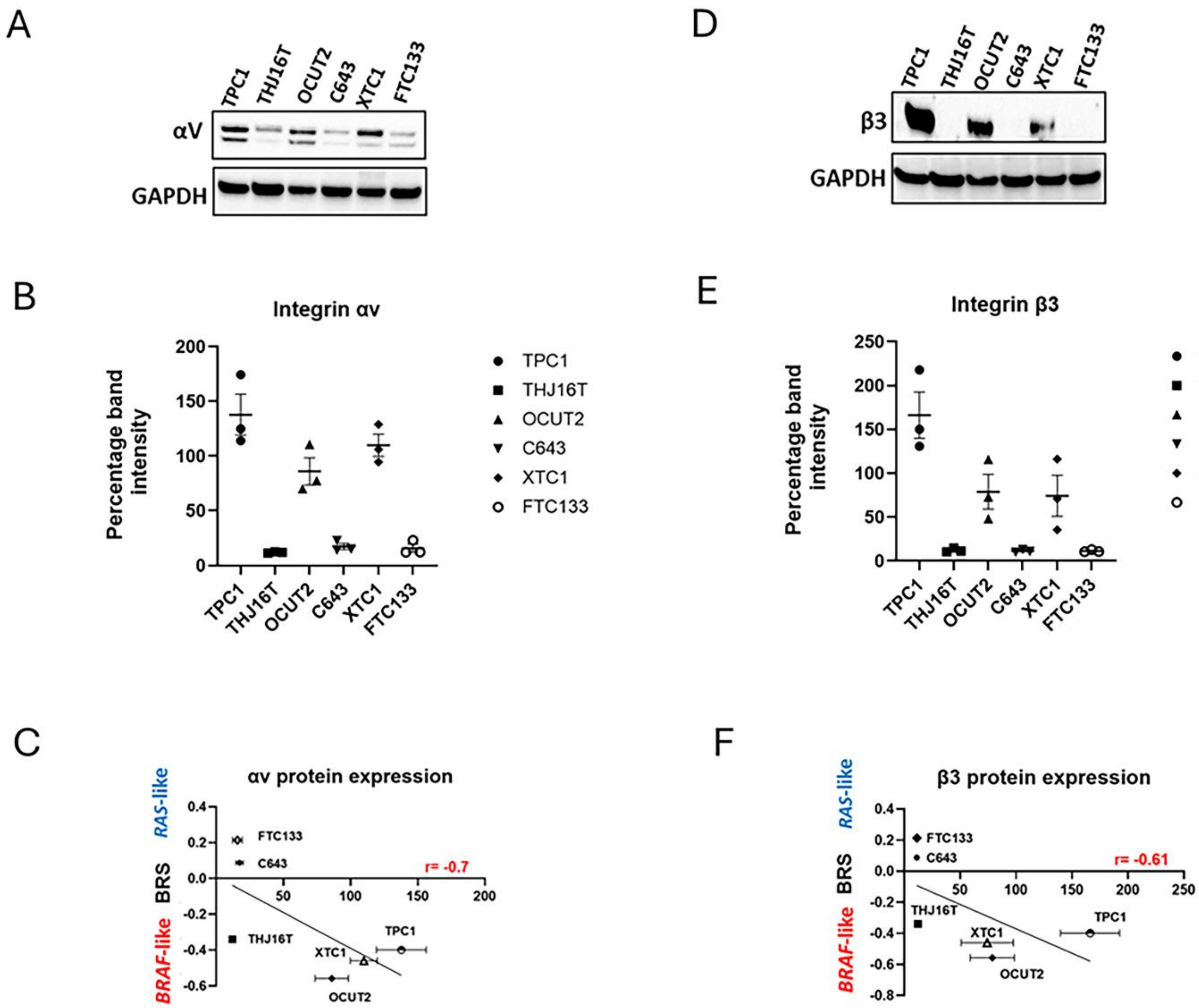

Supplemental Figure 1. (A) Western blot showing the expression of αv proteins in different thyroid cancer cell lines. The highest αv protein expression was found in the TC cell lines characterized by a negative BRS, reflective of more *BRAF*-like transcriptome OCUT2 (BRS = -0.56), TPC1 (BRS = -0.4), and the Hurthle TC cell line XTC1 (BRS = -0.46). **(B)** Quantification of the western blot for αv monomer protein expression (n=3). **(C)** Correlation of α_V and BRS (Pearson, two-tailed): r= -0.7 **(D)** Western blot evaluating the expression of β_3 in different TC cell lines. The highest β_3 protein expression was observed in the TC cell lines with a negative BRS, thus characterized by more *BRAF*-like signature TPC1, OCUT2, and XTC1. **(E)** Quantification of the western blot for β_3 monomer protein expression (n=3). **(F)** Correlation of β3 and BRS (Pearson, two-tailed): r= -0.61. (n=3).

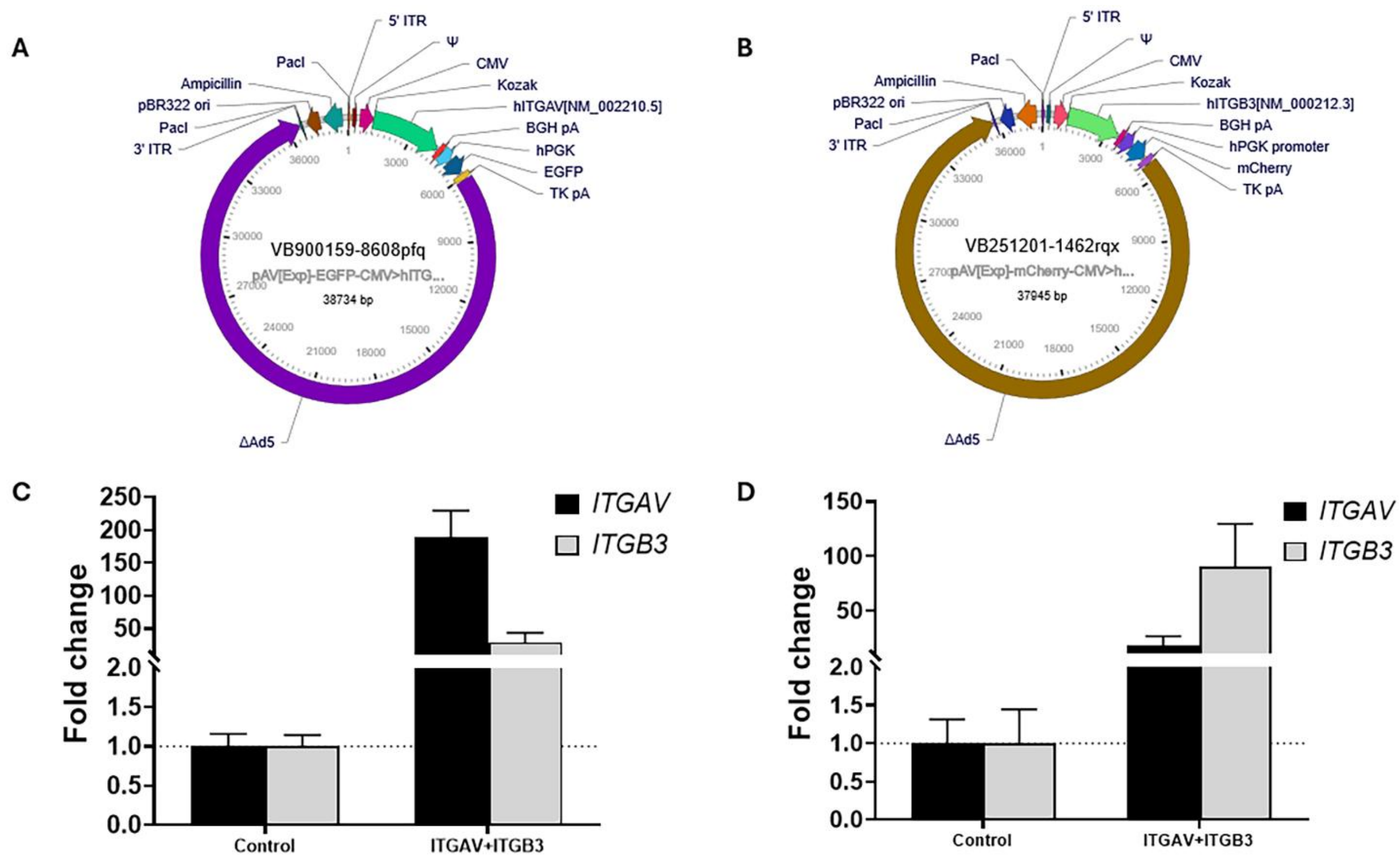

Supplemental Figure 2. Schematic of the custom adenovirus to overexpress the gene **(A)** *ITGAV* and **(B)** *ITGB3*. Real-time qPCR of **(C)** OCUT2 and **(D)** FTC133 cell lines, overexpressed with 10 MOI/cell of adenoviruses of *ITGAV* and *ITGB3* individually for 48 hours, shows successful overexpression of the genes in the cell lines.

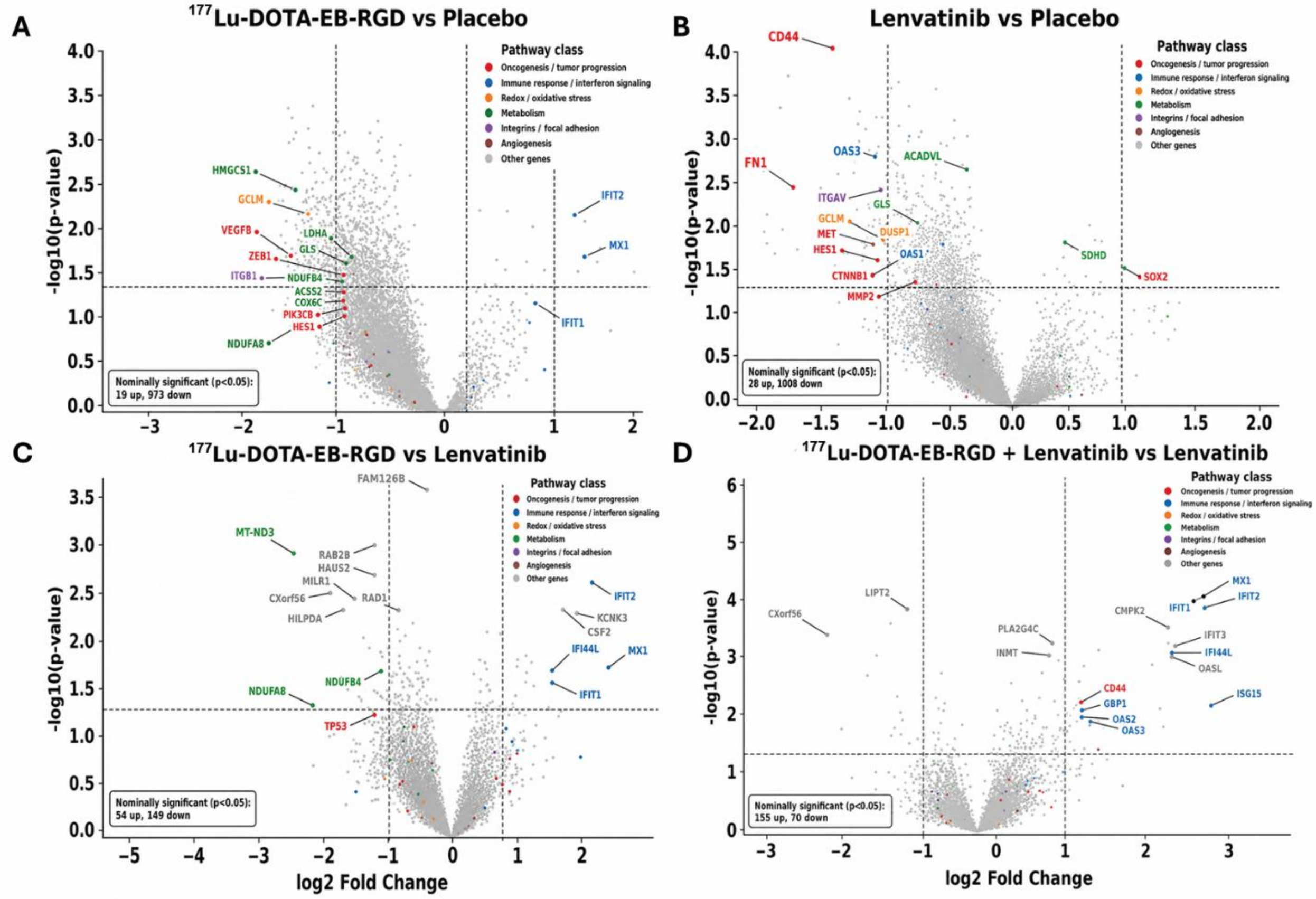

Supplemental Figure 3. RNA sequencing analysis was done on RNA extracted from mouse tumor tissues after termination of the experiment (number of samples: Placebo, n=8; Lenvatinib group L, n=7; ^{177}Lu group, n=8; ^{177}Lu + L, n=6). Data representing the comparisons between **(A)** ^{177}Lu-DOTA-EB-RGD vs Placebo **(B)** Lenvatinib vs Placebo **(C)** ^{177}Lu-DOTA-EB-RGD vs Lenvatinib **(D)** ^{177}Lu-DOTA-EB-RGD + Lenvatinib vs Lenvatinib, is shown. Given the exploratory nature of the analysis and the limited sample size, genes with unadjusted $p < 0.05$ and $|log2FC| \geq 1$ were considered candidates for further validation.

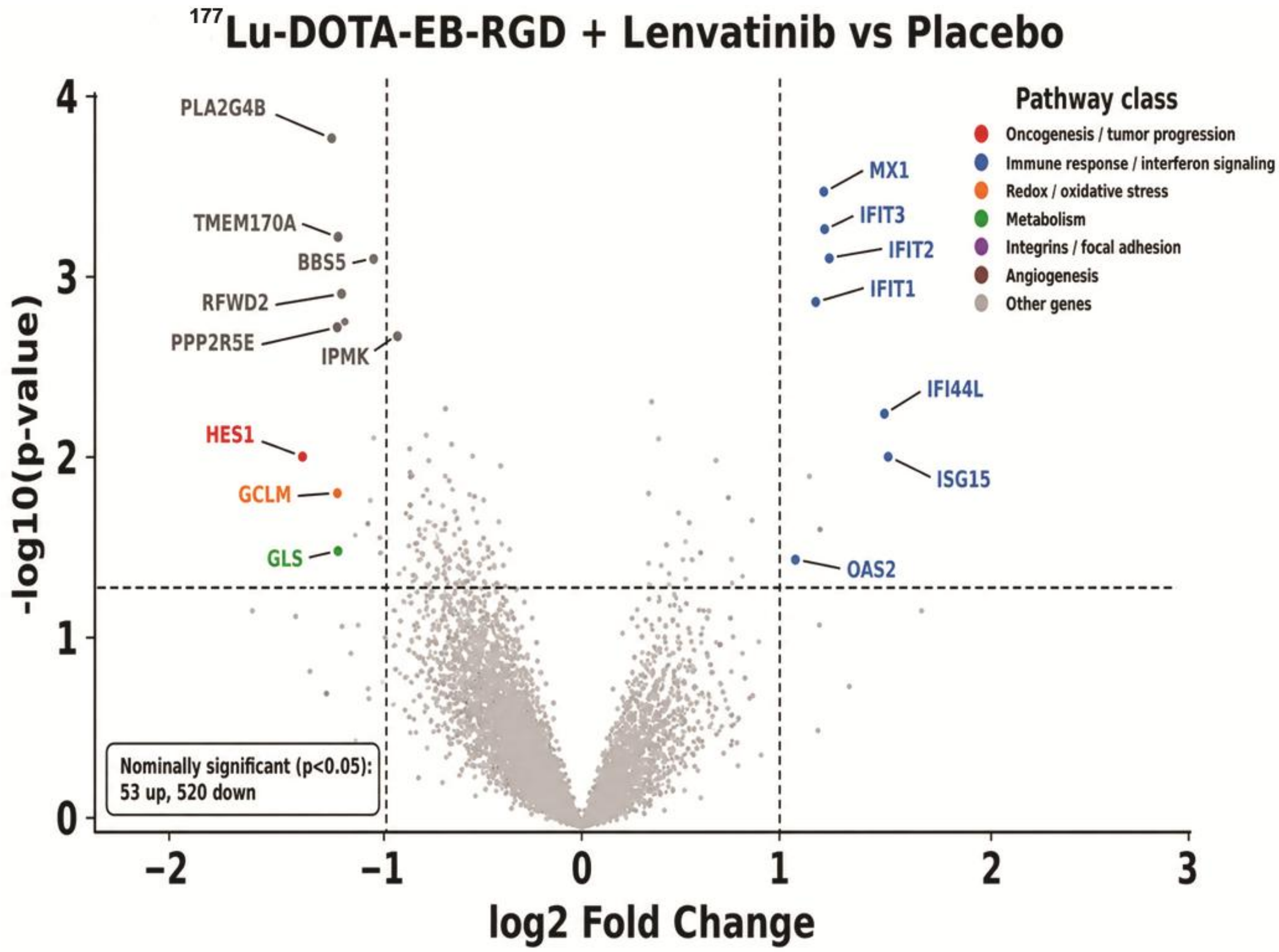

Supplemental Figure 4. RNA sequencing was performed on RNA extracted from mouse tissues after conclusion of the experiment. The figure depicts the comparison between ^{177}Lu-DOTA-EB-RGD + Lenvatinib vs Placebo. Given the exploratory nature of the analysis and the limited sample size, genes with unadjusted $p < 0.05$ and $|log2FC| \geq 1$ were considered candidates for further validation.

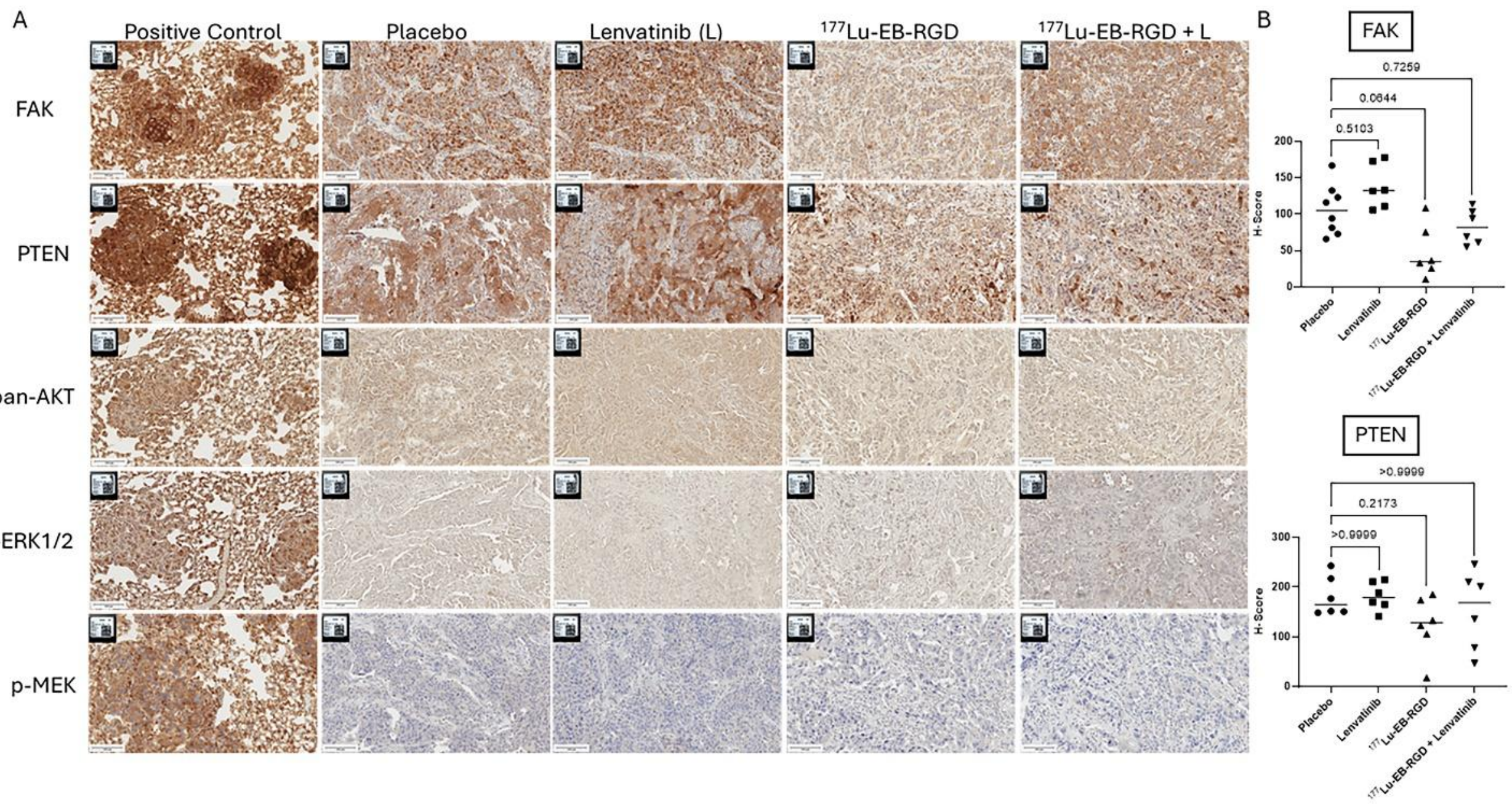

Supplemental Figure 5. (A) Representative images of immunostaining for FAK (focal adhesion kinase; signaling & invasion), PTEN (tumor suppressor), and cell survival and proliferation proteins: pan-AKT, phospho-ERK1/4 (p44/42), and phospho-MEK (Placebo n = 7, Lenvatinib n= 7, ^{177}Lu-EB-RGD n= 6, ^{177}Lu-EB-RGD + Lenvatinib n= 6). (x200 magnification, 100 µm scale bars, identification inset in each image) **(B)** Quantitative analysis of immunostaining for FAK and PTEN in different groups with respect to the control, showing that there is no significant change among the groups (One-way ANOVA, Kruskal-Wallis). No staining for pan-AKT, p-ERK1/2, and p-MEK was observed in comparison to the respective positive controls, revealing no activation of AKT, MEK/ERK signaling in the study cohorts.

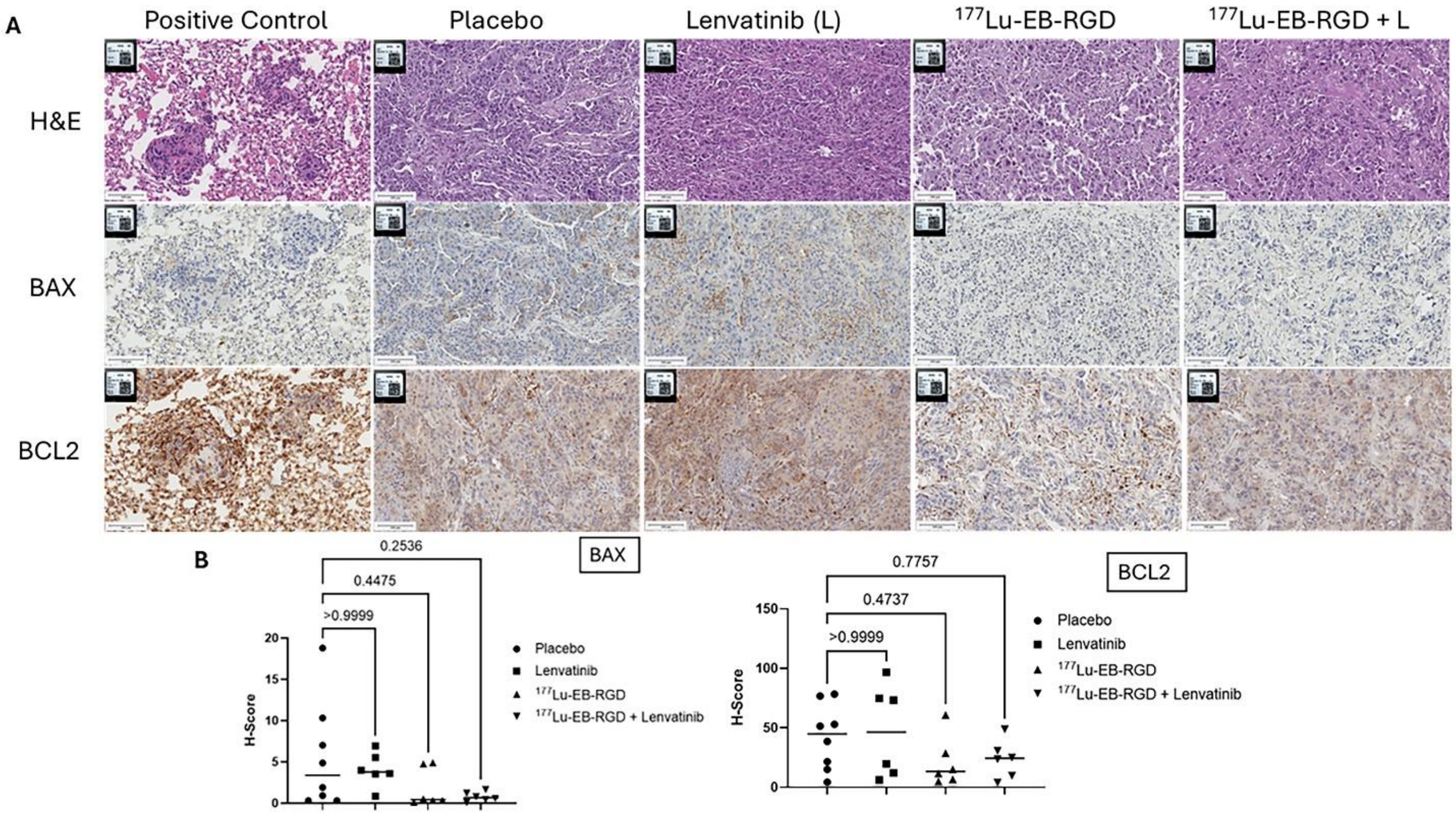

Supplemental Figure 6. (A) Representative images of immunostaining for BAX (pro-apoptotic marker), BCL2 (anti-apoptotic marker), and H&E stain for general morphology (Placebo n = 7, Lenvatinib n= 7, ^{177}Lu-EB-RGD n= 6, ^{177}Lu-EB-RGD + Lenvatinib n= 6). (x200 magnification, 100 µm scale bars, identification inset in each image) **(B)** Quantitative analysis of immunostaining for BAX and BCL2 in different groups with respect to the control, showing that there is no significant change among the groups (One-way ANOVA, Kruskal-Wallis).

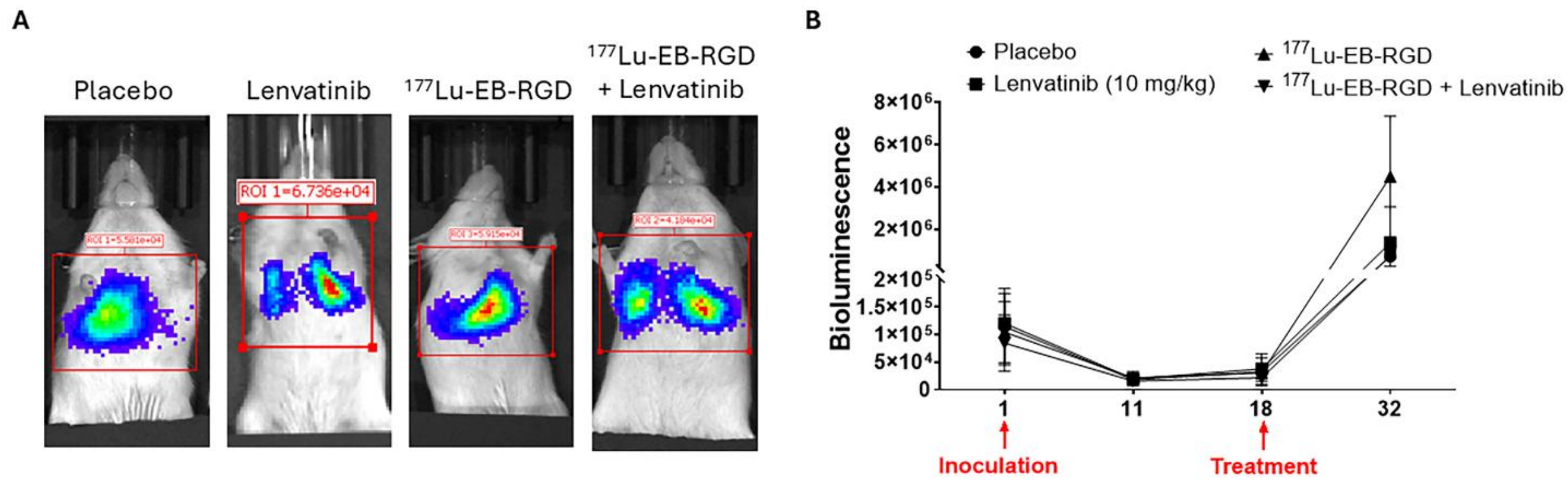

Supplemental Figure 7. (A) Representative images of bioluminescence emission (visualized in IVIS® Lumina II) in NSG mice from different groups intravenously inoculated with 5×10^5 FTC133-Luc cells. **(B)** Comparative range of bioluminescence emitted in mice at different time points of the experiment, including inoculation and start of treatment. No significant change in the tumor burden measured by bioluminescent counts was observed between the treated groups (n=4-7 per group) as compared with the Placebo, $p>0.05$ (Welch's t-test with FDR approach of Benjamini, Krieger and Yekutieli).
