# Supplementary material for: 177Lu-labeled DOTA-EB-RGD as a candidate for thyroid cancer therapy
Source: JCI Insight. 2026 Oct 8;11(19):e200594. doi: 10.1172/jci.insight.200594 (PMC13726428; doi:10.1172/jci.insight.200594)

Full unedited blot for Figure 2B

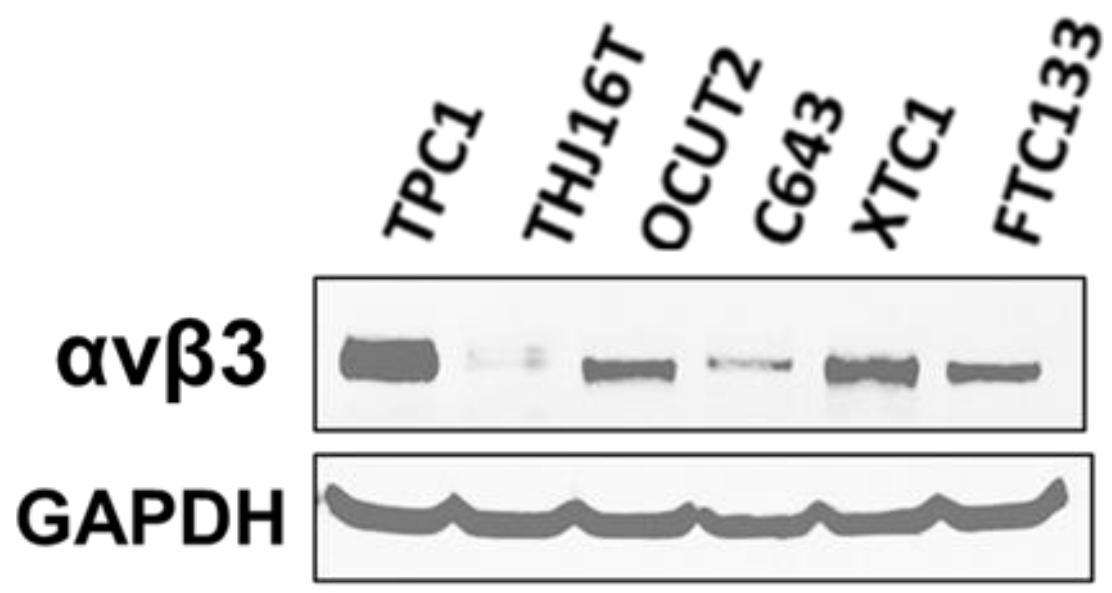

αvβ3

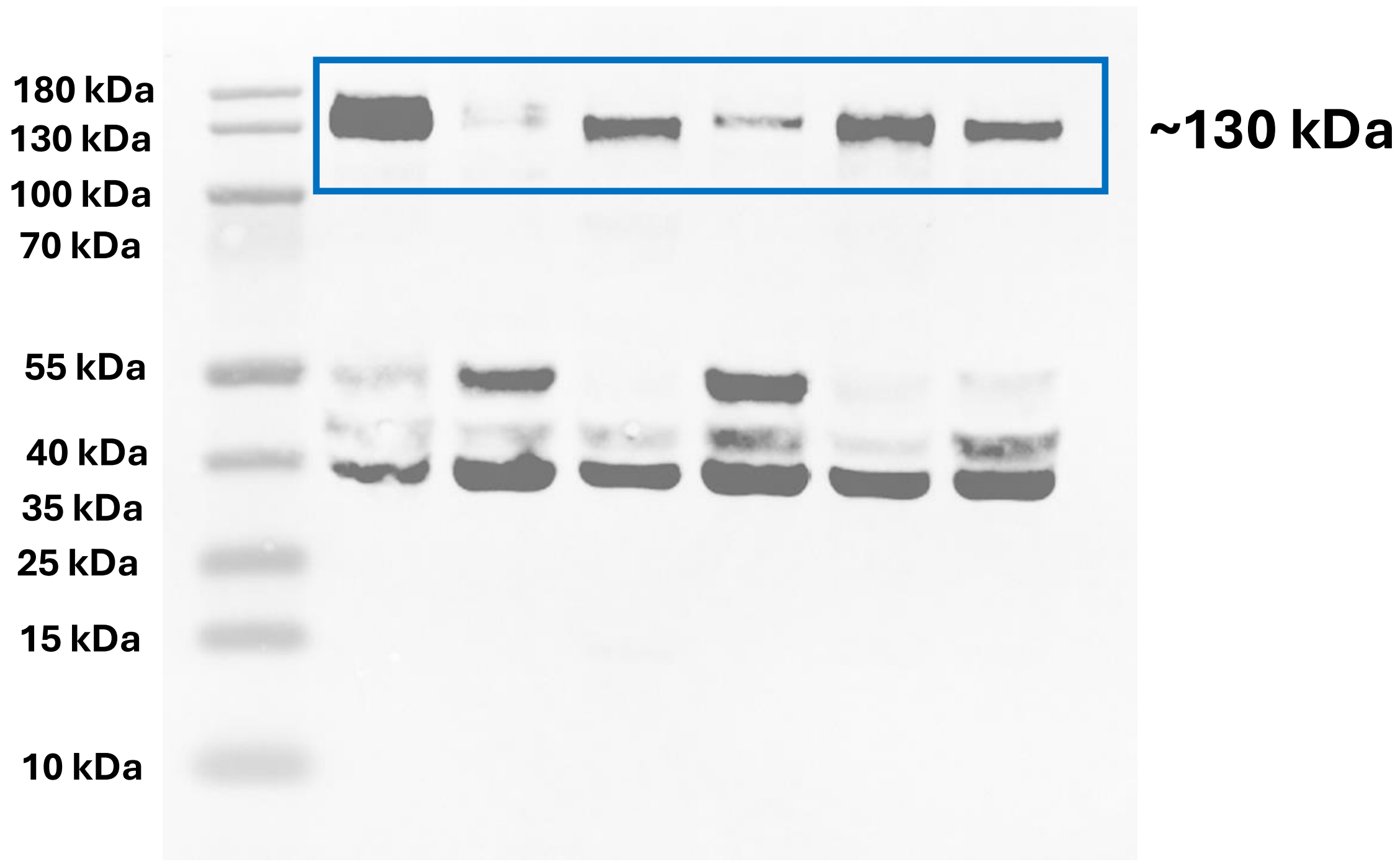

GAPDH

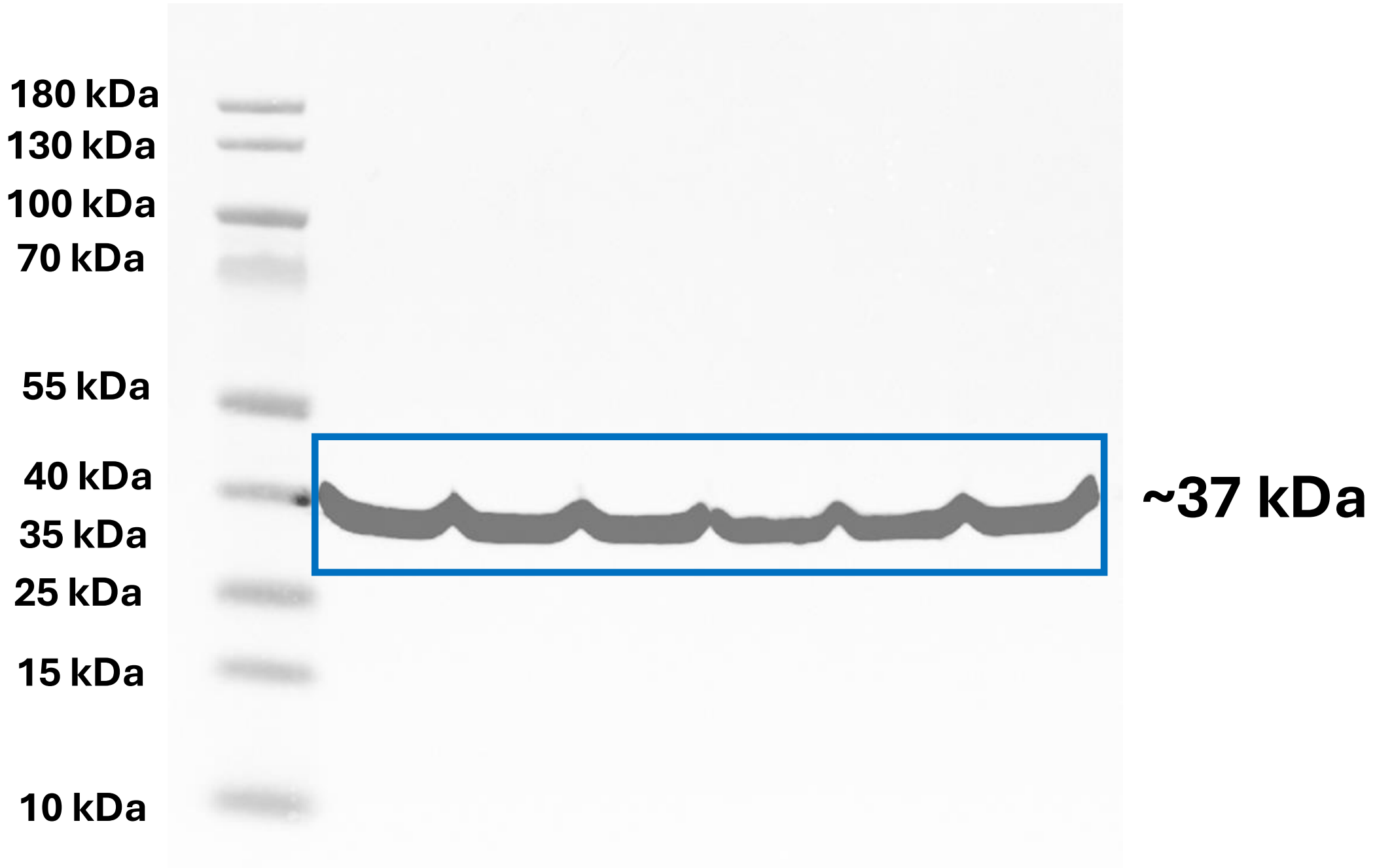

Full unedited blot for Supplemental Figure 1A

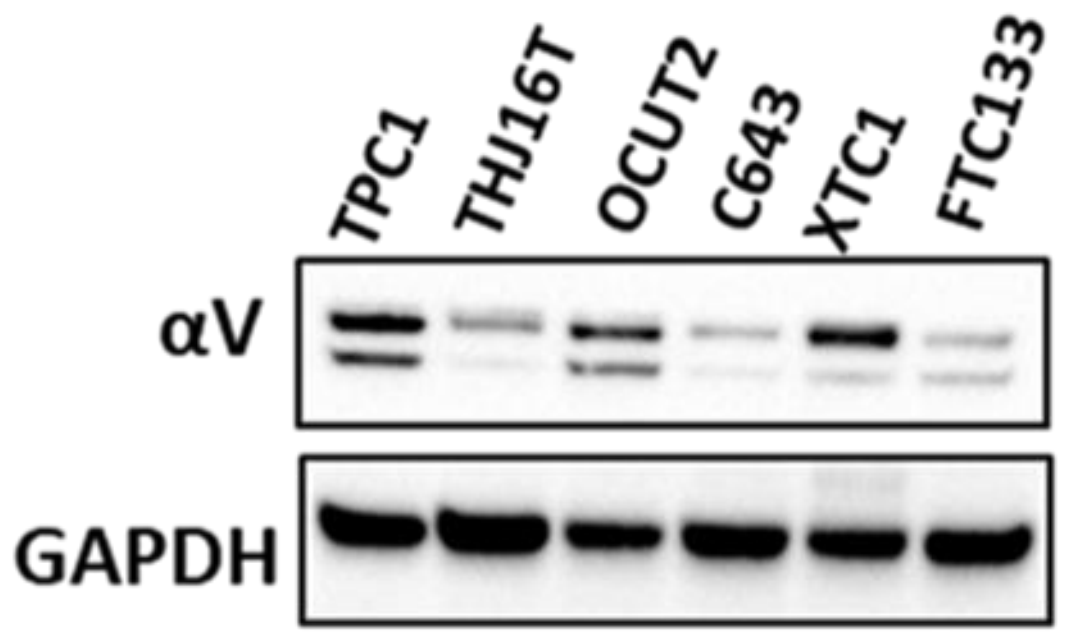

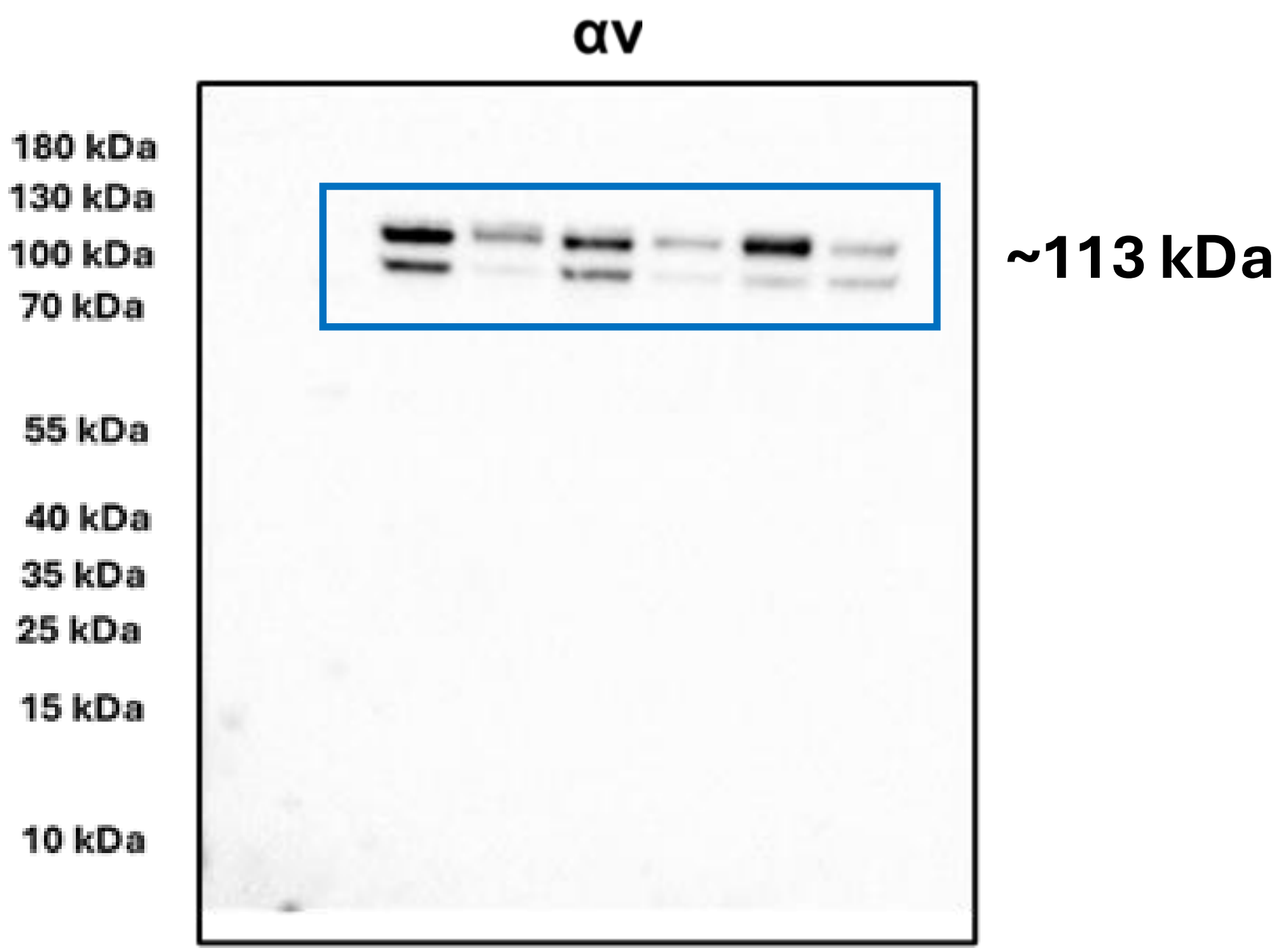

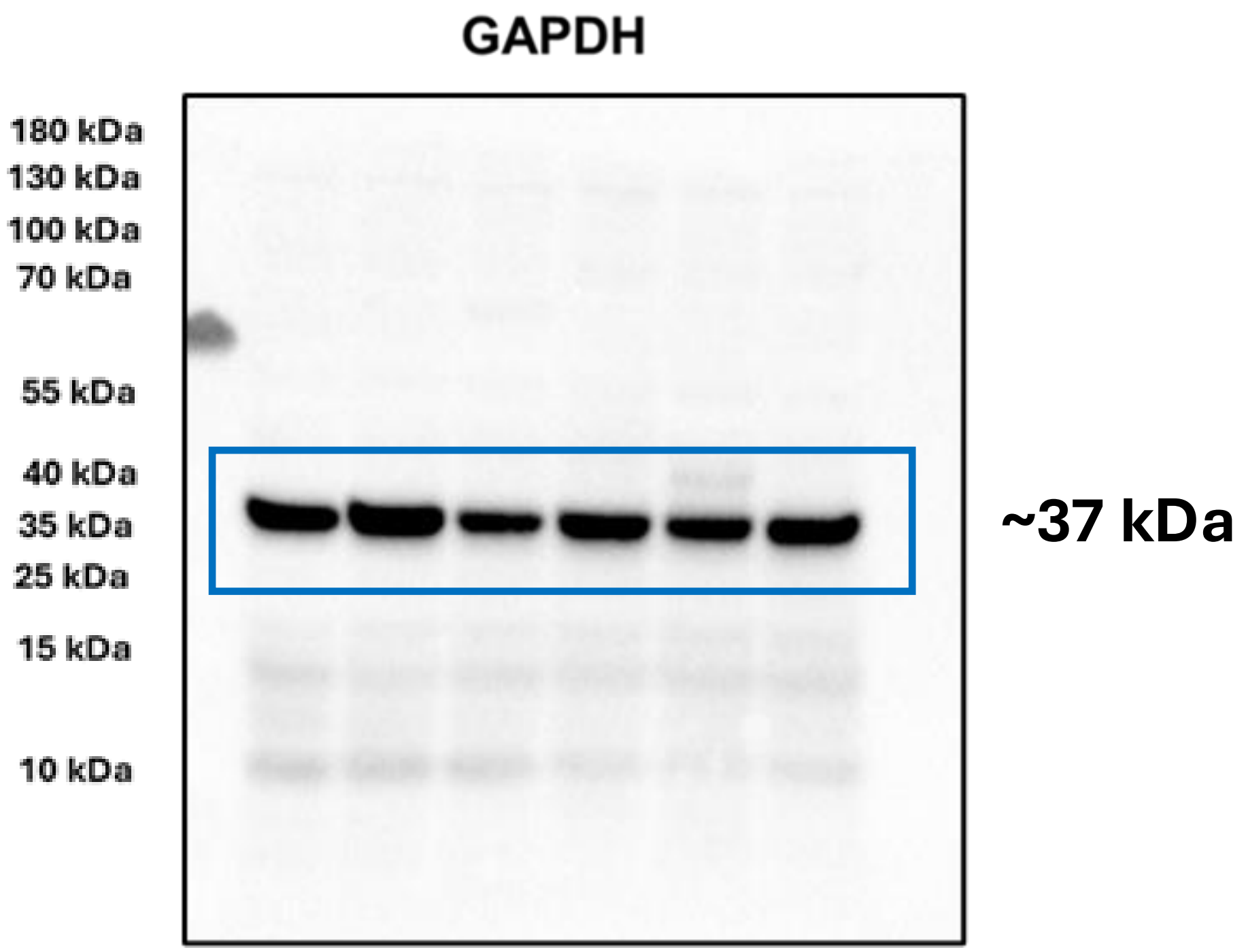

Full unedited blot for Supplemental Figure 1D

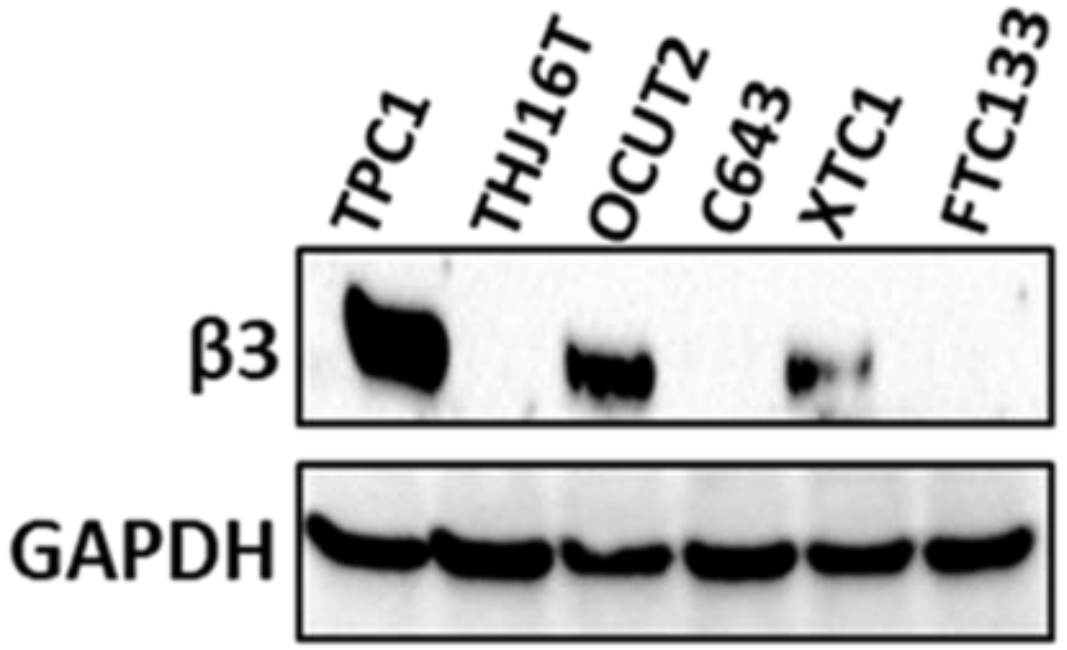

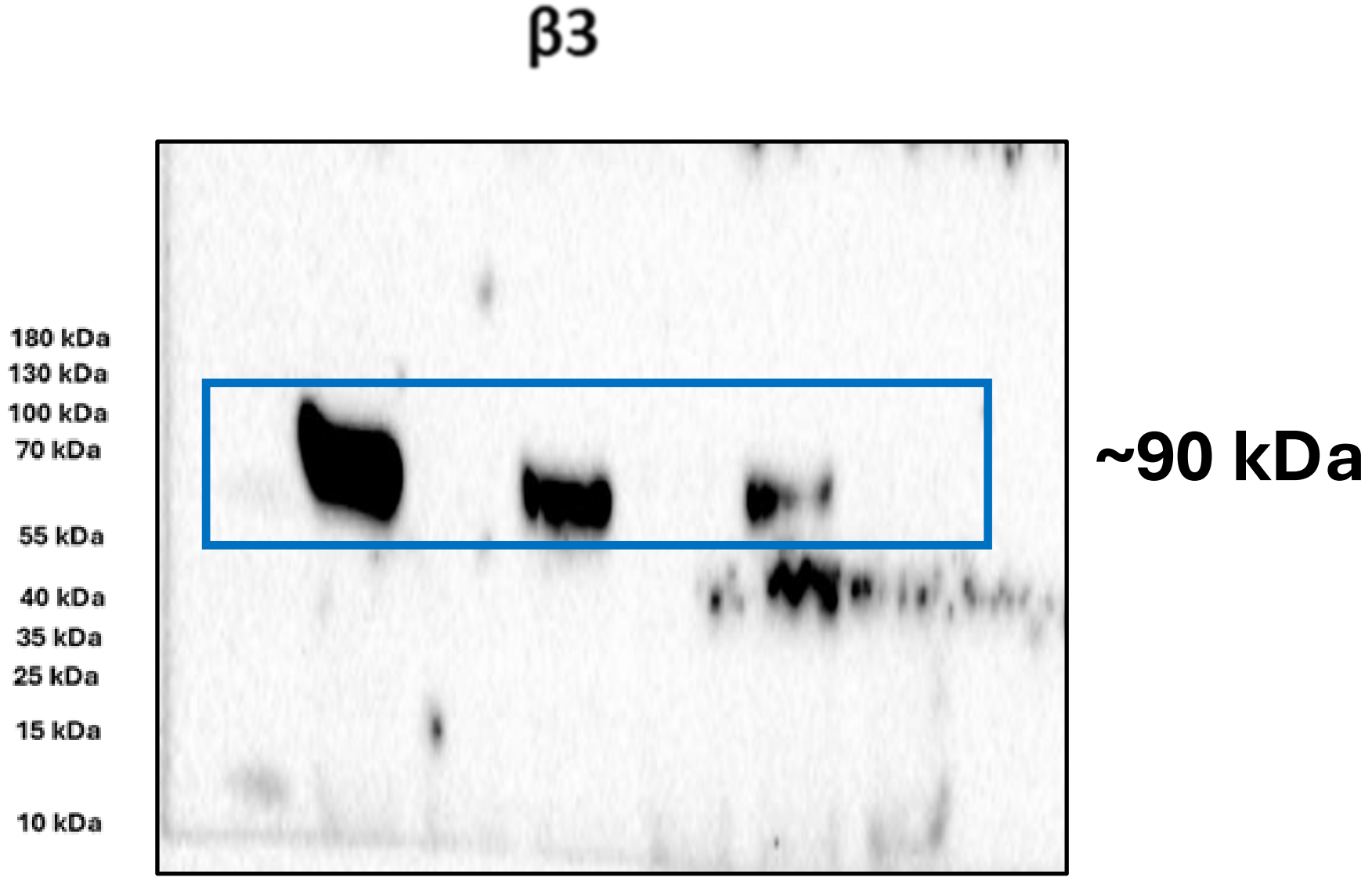

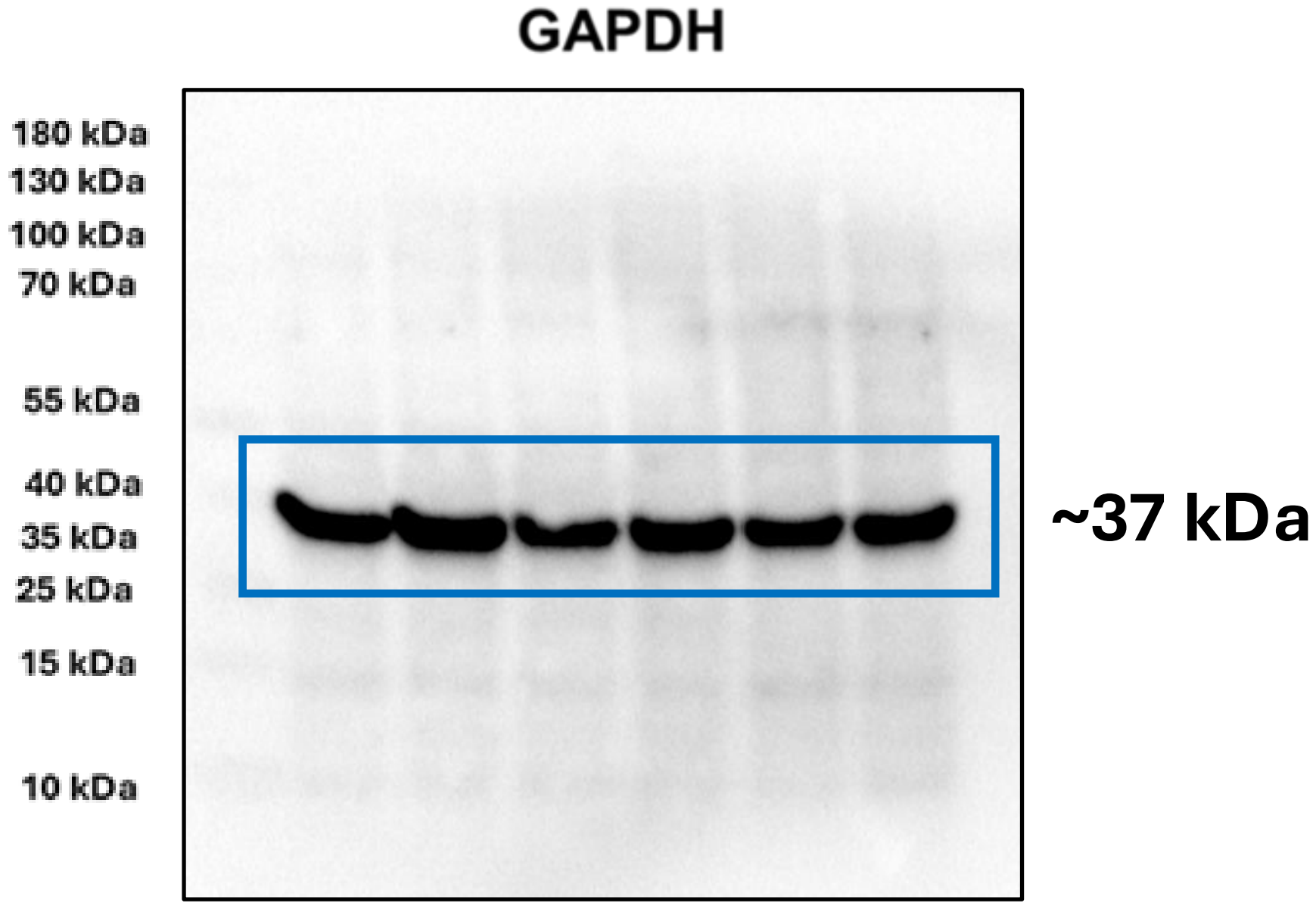

Supplement: Supplementary file 2 [file jciinsight-11-200594-s028.pdf]
